# The nitrone compound OKN-007 delays motor neuron loss and disease progression in the G93A mouse model of amyotrophic lateral sclerosis

**DOI:** 10.3389/fnins.2024.1505369

**Published:** 2024-11-20

**Authors:** Shylesh Bhaskaran, Katarzyna M. Piekarz, Jacob Brown, Brian Yang, Sarah R. Ocañas, Jonathan D. Wren, Constantin Georgescu, Christopher Bottoms, Ashley Murphy, Jessica Thomason, Debra Saunders, Nataliya Smith, Rheal Towner, Holly Van Remmen

**Affiliations:** ^1^Aging and Metabolism Research Program, Oklahoma Medical Research Foundation, Oklahoma City, OK, United States; ^2^Oklahoma Center for Neuroscience, University of Oklahoma Health Sciences Center, Oklahoma City, OK, United States; ^3^Genes & Human Disease Research Program, Oklahoma Medical Research Foundation, Oklahoma City, OK, United States; ^4^Center for Biomedical Data Sciences, Oklahoma Medical Research Foundation, Oklahoma City, OK, United States; ^5^Advanced Magnetic Resonance Center, Oklahoma Medical Research Foundation, Oklahoma City, OK, United States; ^6^Department of Chemistry, University of Prince Edward Island, Charlottetown, Prince Edward Island, Charlottetown, PE, Canada; ^7^Oklahoma City VA Medical Center, Oklahoma City, OK, United States

**Keywords:** amyotrophic lateral sclerosis (ALS), OKN-007, spinal cord, disease progression, cytokine – immunological terms

## Abstract

Our study investigated the therapeutic potential of OKN-007 in the SOD1 G93A mouse model of amyotrophic lateral sclerosis (ALS). The impact of OKN-007, known for its antioxidant, anti-inflammatory, and neuroprotective properties, was tested at two doses (150 mg/kg and 300 mg/kg) at onset and late-stage disease. Results demonstrated a significant delay in disease progression at both doses, with treated mice showing a slower advance to early disease stages compared to untreated controls. Motor neuron counts in the lumbar spinal cord were notably higher in OKN-007 treated mice at the time of disease onset, suggesting neuroprotection. Additionally, OKN-007 reduced microglial activation and preserved reduced neuromuscular junction fragmentation, although it did not significantly alter the increase in astrocyte number or the decline in hindlimb muscle mass. MR spectroscopy (MRS) revealed improved spinal cord perfusion and normalized myo-inositol levels in treated mice, supporting reduced neuroinflammation. While the expression of several proteins associated with inflammation is increased in spinal cord extracts from G93A mice, OKN-007 dampened the expression of IL-1β, IL-1ra and IL-1α. Despite its promising effects on early-stage disease progression, in general, the beneficial effects of OKN-007 diminished over longer treatment durations. Further, we found no improvement in muscle atrophy or weakness phenotypes in OKN-007 treated G93A mice, and no effect on mitochondrial function or lifespan. Overall, our findings suggest that OKN-007 holds potential as a disease-modifying treatment for ALS, although further research is needed to optimize dosing regimens and understand its long-term effects.

## Introduction

Amyotrophic lateral sclerosis (ALS), known as Lou Gehrig’s disease, is a progressive neurodegenerative disease that leads to death of motor neurons in the brain and the spinal cord, causing paralysis and eventually death due to the respiratory failure. There are currently just a handful of treatments with only limited effectiveness approved by the FDA to address the symptoms of this devastating disease (Ansari et al., 2024). The first, approved in 1995, was Riluzole (Rilutek) a compound that acts by inhibiting glutamate release and increases patient survival by approximately 3 months (Jaiswal, 2019). In 2017, Edaravone (Radicava), a free radical scavenger and antioxidant that slows the decline of motor function (a 33% decrease after 6 months of treatment) was approved to specifically address the effects of oxidative stress associated with the disease (Jaiswal, 2019). Other treatments have been approved but still have only limited effectiveness. Importantly, there remains no cure for the disease (Dyer and Smith, 2017) and the search for effective treatments for patients with ALS is critical for the care of these patients.

The success of the free radical scavenger Edaravone as a treatment for ALS supports the potential for other antioxidant compounds as effective interventions. OKN-007 (Oklahoma Nitrone 007; disodium 4-(tert-butyl-imino) methyl benzene-1,3-disulfonate N-oxide, also known as NXY-059 and HPN-07) is a nitrone-based molecule that has antioxidant and anti-inflammatory properties (Maples et al., 2004). While nitrones have been used in free radical-related research since the 1960s (Floyd et al., 2013), OKN-007, the disulfonyl-phenyl derivative of PBN (N-tert-Butyl-*α*-phenylnitrone), has more recently been shown to suppress free radical production in a model of cerebral ischemia (Altinoz and Elmaci, 2018) and in a F98 rat glioma model (de Souza et al., 2015). Nitrone compounds have also been shown to have an anti-inflammatory effect in addition to their antioxidant properties. The OKN-007 parent compound, PBN, prevented microglia activation and loss of NMDA receptors in a kainic acid-induced rat epilepsy model (Floyd et al., 2000; Floyd et al., 2011) and in IL-1β-induced acute brain injury (Fan et al., 2010). OKN-007 is currently being investigated as a treatment for glioblastoma (GBM) (Towner et al., 2019b; Towner et al., 2013). OKN-007 was reported to increase survival and decrease tumor volumes in GBM xenografts (de Souza et al., 2015; Towner et al., 2019b) as well as in liver cancer (Yoon et al., 2018). It is currently in multiple clinical trials for the treatment of glioblastoma (e.g., NCT03649464, NCT03587038, NCT01672463 – clinicaltrials.gov).

Oxidative stress and inflammation are key contributors to ALS-related pathologies (Cunha-Oliveira et al., 2020; Zuo et al., 2021; D’Amico et al., 2013). Because nitrones have anti-inflammatory and free radical scavenging ability, it was proposed that PBN-nitrones might have therapeutic benefit in neurodegenerative disease (Floyd et al., 2013). In support of this, OKN-007 (denominated in the study as HPN-07) in combination with N-acetylcysteine (NAC) was shown to decrease tau immunoreactivity in a rat model of blast-induced traumatic brain injury (du et al., 2016), and had a protective effect on the inner ear and cochlear nucleus in rats exposed to noise (Lu et al., 2014) and in a rat blast injury model (Ewert et al., 2012). Also, OKN-007 (denominated in the study NXY-059) was shown to be neuroprotective in rat model of fluid percussion TBI (Clausen et al., 2008). The potential therapeutic effect of OKN-007 in ALS has not been investigated.

In this study, we investigated whether treatment with OKN-007 can modulate ALS disease onset and/or progression in the SOD1 G93A transgenic mouse model of ALS. Because OKN-007 is a compound with antioxidant, anti-inflammatory and neuroprotective properties, we hypothesized that OKN-007 would preserve alpha-motor neuron (*α*-MN) number and muscle mass, as well as decrease neuroinflammation, which would slow ALS disease progression. To test this, we treated SOD1 G93A mutant mice and wildtype (WT) mice with two different doses of OKN-007 in drinking water that was initiated prior to the onset of ALS symptoms. We measured α-MN number, activation of non-neuronal glial cells, gene transcription and expression of proteins associated with inflammation in the spinal cord at disease onset and during late-stage disease. We also tracked disease onset and progression, as well as effects on skeletal muscle atrophy in mice just prior to disease end stage. Here we report that the treatment with OKN-007 did not delay disease onset, but significantly slowed disease progression. The G93A-treated mice also had a higher α-MN count, as well as lower microglial activation. However, the treatment did not affect muscle mass or survival.

## Materials and methods

### Animals and study design

All experiments were conducted following protocols approved by the Institutional Animal Care and Use Committee at the Oklahoma Medical Research Foundation and the Oklahoma City VA IACUC Committee. G93ASOD1 mice [B6-Tg(SOD1-G93A)1Gur/J(G93AGur1)] were obtained from Jackson Laboratories (Stock number 004435, Jackson Laboratories, Bar Harbor, Maine) and bred on the C57BL/6 J background within the OMRF vivarium establishing a colony for this study. Both male and female mice were used for the initial low dose study. Only female mice were used for experiments at the higher dose. Wild-type C57Bl6J littermates of the same age were used as controls. The mice were housed in a pathogen-free environment with standard chow and water provided *ad libitum*, following a 12 h light/dark cycle. The OKN-007 used for the low dose experiments (150 mg/kg body weight) was generated at OMRF in the laboratory of Dr. Rheal Towner (Piekarz et al., 2022), while the OKN-007 used in the higher dose experiments (300 mg/kg body weight) in Cohorts 1–4 was obtained as a gift from Oblato (NJ, United States). OKN-007 treatment (300 mg/kg) initiated at approximately 30 to 45 days of age (Cohort 4) did not significantly alter lifespan (Supplementary Figure S1).

With the exception of samples used for RNA seq analysis, experimenters were blinded to animal identity during data collection. Sample size was determined based on our experience with these assays in previous similar studies. We routinely use G93A positive males as breeders and females for the experimental cohort (G93A females cannot be used as breeders because the effects of the ALS phenotype occur at a young age). Thus, our experimental mice are predominantly female, with an exception of four male mice included in the NMJ analysis and a small number of male mice (8 male mice: 4 WT AND 4 G93A) that were included in the disease scoring analysis.

### Disease scoring

Weekly weight monitoring and disease scoring were conducted as previously described (Evans et al., 2015). Briefly, the scale of 0–5 was used to describe the disease progression as follows: 0 – non-symptomatic; 1 – hind limb tremor when mouse suspended from tail; 2 – hind limb tremor and difficulty separating hind limbs when suspended by the tail; 3 – difficulty walking; 4 – mouse is unable to walk on all four legs or dragging hind limbs; 5 – mouse unable to right itself within 30s, euthanasia required (end-stage). Disease onset is defined as the age at which the mice first reach a disease score of 1 (hindlimb tremor). Following euthanasia, brain, spinal cord, sciatic nerve, quadriceps, soleus, and gastrocnemius samples were dissected, weighed, and rapidly frozen in liquid nitrogen for subsequent biochemical analysis. Mice showing severe symptoms were euthanized to alleviate suffering.

**Figure 1:**
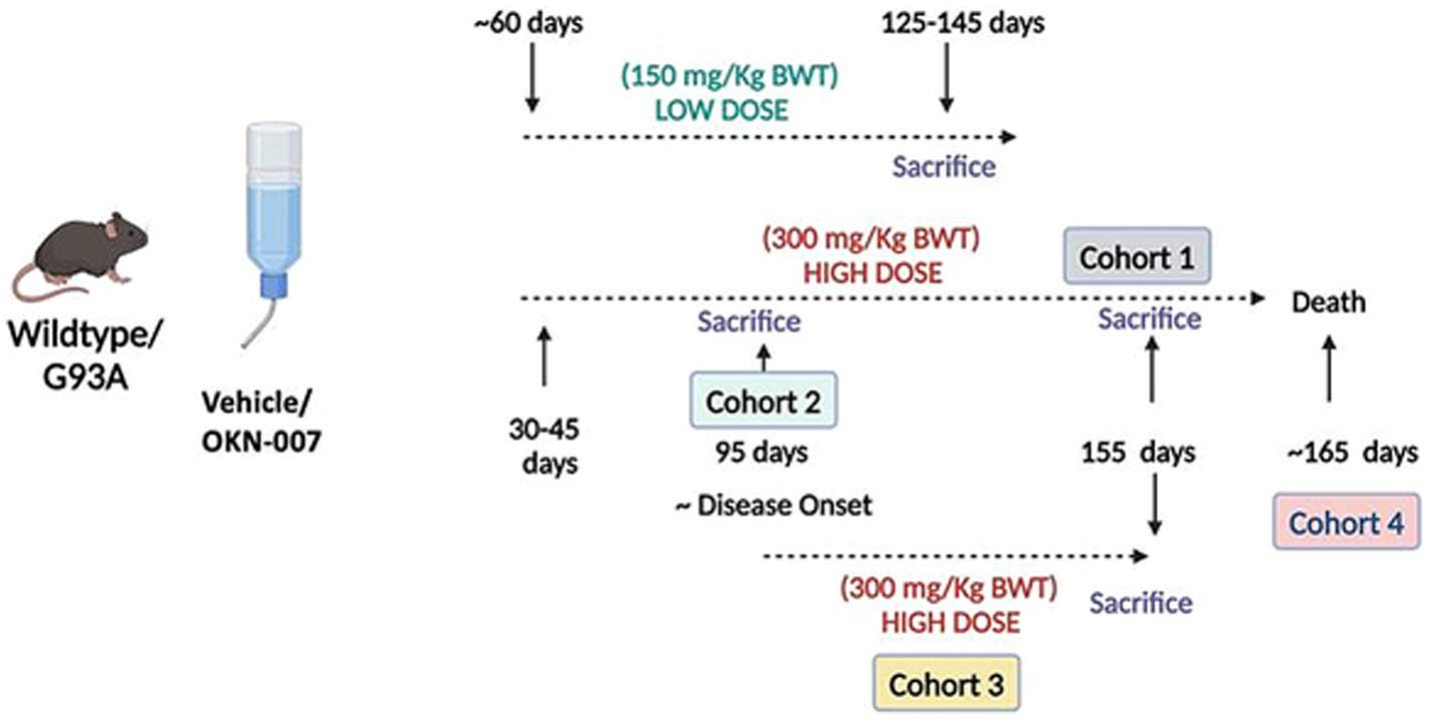
Schematic representation of the experimental design for this study. Five experimental groups were studied: a low dose treatment group (150 mg/kg body weight) beginning at 60 days of age and four separate high dose cohorts (300 mg/kg). In Cohort 1, mice were treated with 300 mg/kg OKN-007 from ~30–40 days of age to 155 days (before disease end stage). In Cohort 2, mice were sacrificed near onset of disease (95 days). Mice in Cohort 3 were treated beginning at 95 days (i.e., onset) and sacrificed at 155 days. Cohort 4 is a lifespan/survival study. Created in BioRender. Van remmen, H. (2022) https://BioRender.com/v74l840

Figure 1 outlines the general experimental design of the study. The study was comprised of five experimental groups. These include: an initial lower dose treatment group (150 mg/kg body weight) provided in drinking water for ~3 months beginning at 60 days of age, and four higher dose cohorts (Cohorts 1–4) at a dose of 300 mg/kg body weight. The dosing at 150 mg/kg was based on a previous study showing that mice tolerated this dose without adverse phenotypes and led to reduced tumor volumes and increased survival in a pediatric glioblasotoma model (de Souza et al., 2015). The positive effects of the 150 mg/kg body weight dose in our initial studies were promising, leading us to ask whether a higher dose (doubling the dose to 300 mg/kg body weight) would be more effective.

In Cohort 1, mice were treated with OKN-007 at 300 mg/kg body weight in drinking water for ~4 months beginning at ~30–45 days of age and sacrificed at 155 days of age (before disease end stage). In Cohort 2, mice were treated beginning at 30–45 days and sacrificed at 95 days (approximate disease onset time). Mice in Cohort 3 were treated beginning at 95 days (approximate age at onset) to mimic treatment in human patients after appearance of disease and sacrificed at 155 days. Cohort 4 is a survival analysis, and the treatment was initiated at 30–45 days as in Cohorts 1 and 2. Survival analysis was conducted using two cohorts of G93A female transgenic mice (21 untreated and 20 OKN-007 treated).

### Immunofluorescence analyses

For these analyses, we followed a previously described protocol (Piekarz et al., 2020) with slight modifications. The spinal cord was carefully collected and the lumbar region (L1–L6) of the spinal cord was fixed overnight in 4% paraformaldehyde (PFA) and subsequently transferred to 30% sucrose in PBS at 4°C overnight. After embedding the spinal cord in optimal cutting temperature (OCT) compound (Sakura Finetek, Torrance, CA) for 1 h, then embedded in a mixture of OCT and TFM (EMS, Hatfield, PA) 1:1 and frozen on dry ice. The following day, the blocks were sectioned on cryotome (Leica; 18 μm thick) and 20 μm transversal sections were mounted on Superfrost Plus Slides (VWR, Radnor, PA). To quantify immunofluorescence, the sections were incubated in a blocking buffer composed of 5% (v/v) normal goat serum (Sigma Aldrich, G9023) and 0.2% (v/v) Triton X-100 (Sigma Aldrich, T8787) in PBS for 1 h at room temperature. Subsequently, the sections were incubated overnight at 4°C in a humidified chamber with specific antibodies targeting motor neurons (anti-NeuN – Cell Signaling #D3S3I, rabbit mAB, 1:500), Microglia cells (anti-Iba1 – Wako #011-27991, goat, 1:250), and astrocytes (anti-GFAP – Abcam, ab7260, rabbit, 1:1000), followed by specific secondary antibody (Invitrogen). The slides were then washed again in 0.3% Triton X-100 in PBS 3× 5 min, and mounted using VectaShield mounting medium (Vestor Laboratories, Inc., Burlingame, CA). The cells were then visualized using Nikon C2 confocal microscope. *α*-MN counts were performed as described previously (Piekarz et al., 2020). Astrocyte and microglia content were established by counting all GFAP- or Iba1-positive cells per ventral horn, respectively. Also, microglia activation was measured by counting the number of non-activated, ramified microglia and expressed both in absolute number and as the percentage of all Iba1-positive cells per ventral horn.

### Determination of motor neuron number

Fluorescent images were obtained using a Nikon confocal microscope to determine the number of motor neurons. As described above for astrocyte and microglial analyses, sections analyzed were from the lumbar (L1–L6) spinal cord. Five sections per animal/group were imaged using a × 20 objective. Motor neurons, identified as NeuN positive cells situated within the ventral horn, were quantified based on size, morphology, and location. The average count per group (*n* = 5) were determined. The analysis of images was performed in a blinded manner with respect to sample identity. Additionally, IBA-1 and GFAP positive cells representing microglia and astrocytes, respectively, were counted independently.

### NMJ

Neuromuscular junction morphology and fragmentation was assessed as described previously (Jang et al., 2010; Xu et al., 2021). Briefly, confocal microscopy was utilized to examine the morphology of neuromuscular junctions (NMJs). Gastrocnemius muscle sections were carefully prepared in cold PBS in a petri dish, ensuring the preservation of fiber directions while removing fat and connective tissues, as previously described. These muscle samples were then placed in a 24-well plate containing 10% STUmol in deionized water (Poly Scientific R&D) for 1 h to fix the tissue with gentle shaking. After fixation, the tissues underwent three 5 min washes in PBS at room temperature, followed by permeabilization in 2% Triton in PBS for 30 min on a shaker. Subsequently, the tissues were placed in a blocking buffer composed of 4% BSA, 1% Triton, and 5% serum matching the host of the secondary antibody and were blocked overnight at 4°C. Primary antibodies, including 1:50 SV2 (DSHB) for nerve terminals and 1:50 2H3 (DSHB) for neurofilaments, were added after blocking. The tissues were then incubated overnight at 4°C, followed by 6 washes of 30 min each in PBS at room temperature. Next, the tissues were incubated with secondary antibodies: 1:1,000 BTX-Alexa 488 (Invitrogen) and 1:250 goat anti-mouse Cy3, overnight at 4°C. Following this, the tissues underwent another 6 washes of 30 min each with PBS. The tissues were then transferred onto slides, mounted with a suitable mounting medium, and sealed with nail polish. Images of the NMJs were captured using a Nikon confocal microscope at 20x magnification. Z-stacks were obtained to visualize the 3D structure of intact NMJs. The total thickness of optical sections ranged from 20 to 60 μm, with a stack interval set at every 1 to 2 μm, resulting in approximately 20 to 30 images per stack.

Analysis of the NMJ area involved quantifying the area occupied by each individual labeled acetylcholine receptor (AChR), with only the NMJs facing forward considered for analysis. The level of fragmentation was determined by counting the fragmented pieces of each AChR. NMJs with five or more pieces per junction were classified as fragmented. The denervation score was assessed as follows: score 0 indicated no denervation, with AChRs fully overlapping nerve terminals; score 1 indicated partial denervation, with partial overlap of AChRs and nerve terminals; and score 2 indicated complete denervation, with little or no overlap between AChRs and nerve terminals. For NMJ area analysis, all images from this section were evaluated by outlining each individual forward-facing NMJ, calculating the actual area in μm^2^ using ImageJ software, and importing the data into GraphPad Prism 8 for statistical analysis.

### *In vivo* MR techniques

Mice were anesthetized and positioned in an MRI cradle. A 30 cm horizontal bore Bruker Biospin magnet operating at 7 T (Bruker BioSpin GmbH, Karlsruhe, Germany) was used to obtain imaging and biophysical parameters (diffusion, perfusion coefficients). A BA6 gradient set and mouse head coil were used to perform all MRI experiments as previously described (Zalles et al., 2020; Towner et al., 2021). The perfusion imaging method (ASL) was used as previously described (Zalles et al., 2020). Perfusion maps were obtained from a single axial slice of the lumbar spinal cord region. Five regions of interest (ROIs) were manually outlined in the image field-of-view. Relative tissue blood flow (rTBF) values were calculated using Paravision software (Bruker Biospin).

### Diffusion-weighted imaging

Diffusion-weighted images were obtained using a single-shot echo-planar encoded imaging sequence with a repetition time of 1,325 ms, an echo time of 63 ms, and a flip angle of 90°, as previously described (Bozza et al., 2010; Towner et al., 2012). Geometric distortions inherent to this encoding scheme applied at an operating proton frequency of 300 MHz were greatly diminished by careful shimming of the volume of interest using the “fast automatic shimming technique by mapping along projections” method. Diffusion-sensitizing gradients (7 ms) were applied along the rostro-caudal axis, separated by 14 ms and located at symmetric positions on either side of the refocusing pulse. Five images were obtained with different gradient gains, resulting in b values of 0, 400, 800, 1,200, and 1,600 s/mm^2^. For each voxel, the signal intensities measured at different b values, S(b), were numerically fitted against the model, S(b) = S(b = 0) e(−b.ADCx). All calculations were performed using Paravision software (Bruker Biospin). Five ADC values were obtained from the image field of view.

### MR spectroscopy

^1^H–MRS was acquired using a PRESS (Point Resolved Spectroscopy) sequence as previously described (Piekarz et al., 2020). More specifically, a TE of 24.0 ms, a TR of 2500.0 ms, 256 averages, and a spectral width of 4,006 Hz, were used. A non-suppressed MR spectrum was acquired beforehand by applying eddy-current correction to maximize signal intensity and decrease the peak linewidths. Water was suppressed with a VAPOR (variable power radio frequency pulses and optimized relaxation delays) suppression scheme. In all cases, the peak width (full width at half maximum) of the water peak was less than 30 Hz following localized shimming, which was conducted by using first- and second-order adjustments with Fastmap. A cubic voxel of 1.5 × 2.0 × 1.5 mm^3^ was positioned in the mouse spinal cord. To analyze the MRS data, an in-house Mathematica program was used (version 8.0.4.0, Wolfram Research, Champaign, IL, United States). The spectra were scaled in ppm by calibrating against the water peak (4.78 ppm). The major metabolic peaks were identified as Lipids-CH3 at 0.9 ppm, Lipids/Lactate at 1.3 ppm, N-acetylaspartate (NAA) at 2.02 ppm, glutamate (Glu) at 2.3 ppm, creatine (Cr) at 3.02 ppm, choline (Cho) at 3.22 ppm, taurine (Tau) at 3.43 ppm, and myo-inositol at 3.57 ppm. The peak area measurements of each metabolite were used to calculate ratios against Cr (metabolite/Cr).

### Cytokine array

The inflammatory cytokines in mouse lumbar spinal cord were measured using a Protein Profile Array (Mouse Cytokine Array Panel A #ARY006, R&D Systems, Minneapolis, MN) following the manufacturer’s protocol. In short, mice were perfused with cold PBS, and the lumbar region of the spinal cord was extracted and homogenized in PBS with a protease inhibitor cocktail. 200 μg protein of spinal cord extract was utilized for the assay (Bhaskaran et al., 2023).

### RNA seq analysis

RNA was isolated from the spinal cord of two groups of mice: one group treated and the other untreated, consisting of both wild type and G93A mice. Each sample weighed 25 mg, and the TRIzol reagent (Invitrogen, CA, United States) was used according to the manufacturer’s instructions for the extraction process. Subsequently, the extracted RNA samples were submitted to the OMRF Clinical Genomics Center for further processing. To ensure compliance with quality standards, the concentration and integrity of the RNA were evaluated using Agilent Tapestation. Libraries were constructed using the TruSeq Stranded mRNA Library Kit from Illumina, and the resulting libraries were sequenced on an Illumina NextSeq 500 platform. Raw sequencing reads (in a FASTQ format) were trimmed using Trimmomatic (Bolger et al., 2014) to remove any low-quality bases at the beginning or the end of sequencing reads as well as any adapter sequences. Trimmed sequencing reads were aligned to the *Mus musculus* genome reference (GRCm38/mm10) using STAR v2.4.0 h (Dobin et al., 2013). Gene-level read counts were determined using HTSeq v0.5.3p9 (Anders et al., 2015) with the GENCODE Release M10 (GRCm38) annotation. edgeR (Robinson et al., 2010) was utilized for read-count normalization and differential gene expression analyses.

## Supplemental methods

### Contractile force measurement

Contractility measurements were conducted on isolated extensor digitorum longus (EDL) muscles using a 1200A *in-vitro* test system from Aurora Scientific Inc. (Aurora, ON, Canada), following our previously described methods (Xu et al., 2022b; Xu et al., 2021). The EDL muscle of the hind limb was chosen for analysis due to its well-documented response to oxidative stress associated with neuromuscular diseases. Each muscle was individually attached to a model 300C servomotor (Aurora Scientific Inc.) and immersed in a water bath filled with oxygenated (95% O2, 5% CO2) Krebs-Ringer solution containing the following concentrations in millimolar (mM): 137 NaCl, 5 KCl, 1 MgSO_4_, 1 NaH_2_PO_4_, 24 NaHCO_3_, and 2 CaCl_2_. The temperature of the bath was maintained at 32°C.

Computer-controlled stimulation was applied using a model 701C stimulator (Aurora Scientific Inc.) through platinum plate field stimulus electrodes positioned on either side of the muscle at supramaximal voltage, with a pulse width of 0.2 ms, and at the optimal length for producing twitch force (i.e., Lo). The time taken to reach maximal force from the baseline, known as time to peak (TTP), and the time taken to achieve 50% relaxation from the peak, known as half relaxation time (RT ½), were measured for both twitch and tetanic contractions. Force-frequency curves were generated by applying stimulation frequencies ranging from 1 to 300 Hz. The fatigue protocol involved the repeated application of 300 ms trains of 150 Hz stimuli every 5 s for a total duration of 200 s. All data were recorded and analyzed using commercial software provided by Aurora Scientific Inc. (DMC and DMA). To calculate specific force (N/cm2), muscle length and weight were measured as previously described (Xu et al., 2021).

### Mitochondria function: simultaneous high-resolution respirometry and fluorometry measurements

To measure the oxygen consumption rate (OCR) and the rate of mitochondrial hydrogen peroxide (H_2_O_2_) production, we utilized the Oxygraph-2 k (O2k) system as we have previously described (Xu et al., 2022a). The experiments were conducted using permeabilized fibers in buffer X media (7.23 mM K2EGTA, 2.77 mM CaK_2_EGTA, 20 mM imidazole, 0.5 mM DTT, 20 mM taurine, 5.7 mM ATP, 14.3 mM PCr, 6.56 mM MgCl_2_-6H_2_O, and 50 mM K-MES (pH 7.1)), saponin (30ug/ml) was added followed by three washes for 5 min washes in washing buffer (buffer Z) containing 105 mM K-MES, 30 mM KCl, 10 mM K_2_HPO_4_, 5 mM MgCl_2_-6H_2_O, bovine serum albumin (BSA; 0.5 mg/mL), and 0.1 mM EGTA (pH 7.1) at a temperature of 37°C. The measurement process involved the reaction between hydrogen peroxide and Amplex UltraRed, catalyzed by horseradish peroxidase (HRP), resulting in the production of the fluorescent compound resorufin. A standard curve was established daily to convert the fluorescent signal into nanomolar H_2_O_2_ concentration, with background resorufin production subtracted from each measurement.

To evaluate the rates of respiration and H_2_O_2_ production, we employed sequential additions of substrates and inhibitors. These additions included glutamate (10 mM), malate (2 mM), ADP (2.5 mM), succinate (10 mM), and rotenone (0.5 μM) + Antimycin A (1 μM). To ensure non-mitochondrial respiration does not influence the respiration measurements presented in this manuscript, fiber bundles were treated with rotenone + antimycin A and values were subtracted from other respiratory measurements. The resulting OCR and H_2_O_2_ production data were further normalized by the wet weight of the muscle (in milligrams) to ensure accurate comparison between samples.

### Effect of OKN-007 on disease progression in the G93A mouse model of amyotrophic lateral sclerosis

**Figure 2:**
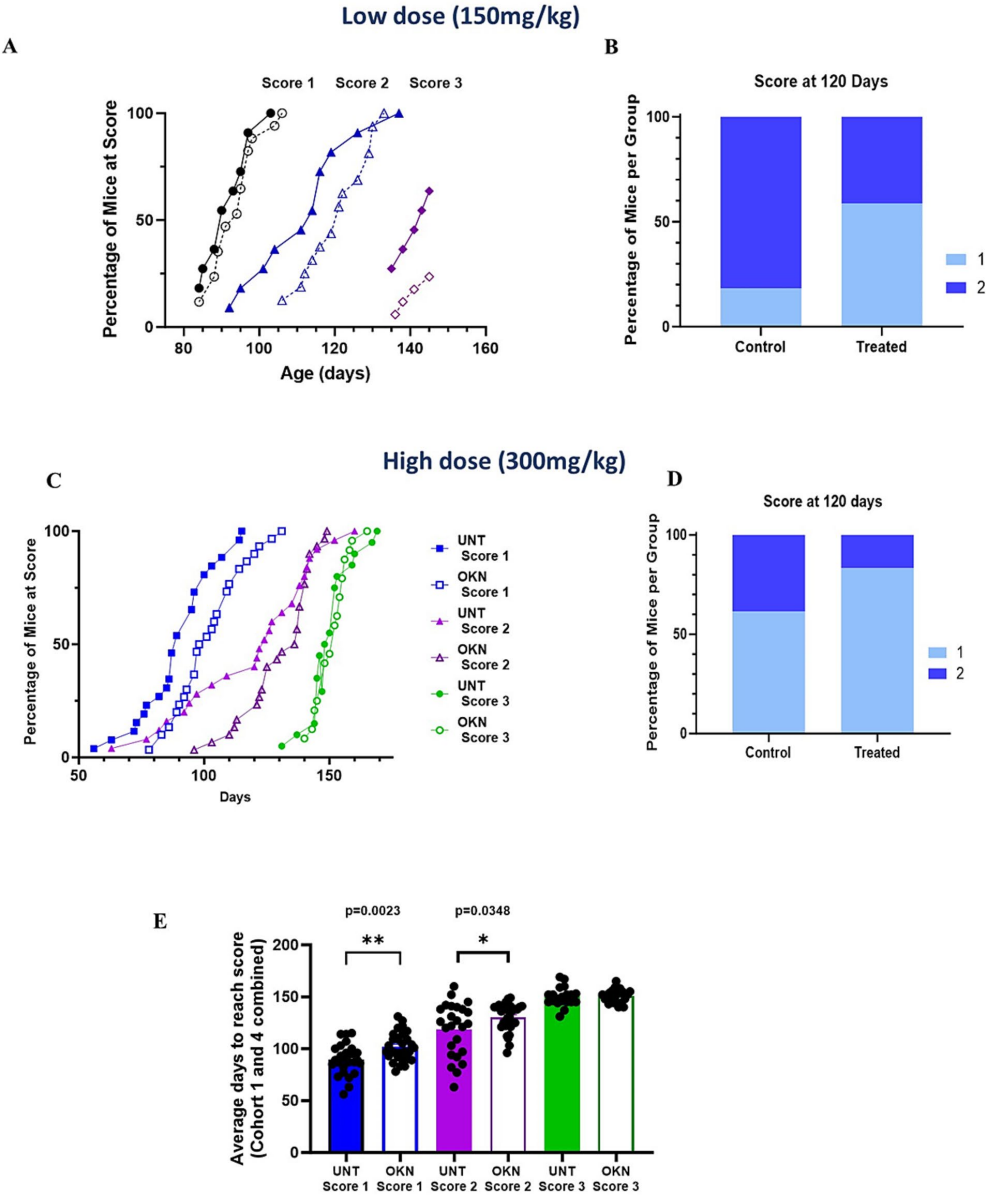
Treatment with OKN-007 delays disease progression in G93A mice. G93A mice (mixed male and female) were subjected to a schedule of either OKN-007 or H2O (vehicle) for control animals starting at 60 days of age and continued until 145 days of age. Disease progression was assessed on a scale of 0–5, where 0 represents non-symptomatic and 5 indicates severe symptoms. The detailed scale is as follows: 0 – non-symptomatic; 1 – hind limb tremor when mouse suspended from tail; 2 – hind limb tremor and difficulties separating hind limbs when suspended from tail; 3 – difficulties walking. **(A)** The percentage of mice reaching a score of 1 to 3 in both untreated (closed symbols) and OKN-treated (open symbols) groups is graphically represented as a function of age. **(B)** Shows the total percentage per group at 120 days of age depicted as a histogram. **(C,D)** G93A mice were treated with a higher dose of OKN (300 mg/kg), administration starting at 30 to 45 days of age and continued until 155 days. The data shown represent data combined from mice in Cohorts 1 and 4 treated (*n* = 30) and untreated (*n* = 26) mice. **(E)** Shows the cumulative G93A disease scores of female mice [untreated (*n* = 26) and treated (*n* = 30)] from cohorts 1 and 4 in the high dose treatment group (300 mg/kg). There is a notable shift in disease progression that is significant for disease score 1 (*p* = 0.0023) and disease score 2 (*p* = 0.0348) between untreated and treated mice. The asterisk (*) highlights significant distinctions in G93A mice between the treated and untreated groups at 100 and 120 days, as determined by unpaired *t*-test (*p* < 0.05). The data are presented as mean ± SEM.

Disease progression was delayed in the G93A mice in response to both the lower (Figures 2A,B) and higher dose treatments (Figures 2C,D). In response to the lower dose (150 mg/kg OKN-007), both treated and untreated mice became symptomatic (reaching a score of 1) beginning close to 95 days. Although the median age to reach a score of 1 was not different, as time progressed, the treated and untreated groups diverged. Low-dose OKN-007 treatment significantly slowed disease progression, and treated mice reached a score of 2 at the median age of 121 days, whereas untreated mice progressed to stage 2 at a median of 113 days (*p* = 0.0375) (Figure 2A). The mean age at which mice progressed to stage 3 (difficulty walking) was approximately 140 days and was not different between groups. One untreated mouse reached a score of 4 (unable to walk or dragging their limbs), while none of the treated mice progressed to that stage by the time of sacrifice. Figure 2B illustrates the percentage of mice that have reached a score of 1 or 2 at 120 days of age. While 18 percent of the untreated mice were at a score of 1 and 82% were at a score of 2 at this age, only 44% of the OKN-007 treated mice reached a score of 2 at this age and 59% were still at a score of 1. Figure 2C shows a similar effect in response to the higher dose of OKN-007 (300 mg/kg) where treatment was started at 30–45 days of age. Data were combined from Cohorts 1 and 4 for data shown in Figures 2C–E. Disease progression was delayed in the treated mice for scores of 1 and 2 (open symbols) and at 120 days of age only 17% of the OKN-007 treated mice had reached a score of 2 compared to 39% of the untreated mice (Figure 2D). As in the lower dose cohort, there was no difference at stage 3 between groups. The number of days needed to reach a score of 1, 2 or 3 in mice from Cohorts 1 and 4 at the higher dose (Cohort 4 is a lifespan/survival cohort) are shown in Figure 2E. There was a significant difference observed in the time required for untreated versus OKN-007 treated mice to reach a disease score 1 (*p* = 0.0023) and disease score 2 (*p* = 0.0348). Together, these results show that both low and high dose treatment with OKN-007 significantly impacted disease progression in G93A mice at earlier disease stages.

### OKN-007 delays the loss of *α*-MNs in G93A mice

**Figure 3:**
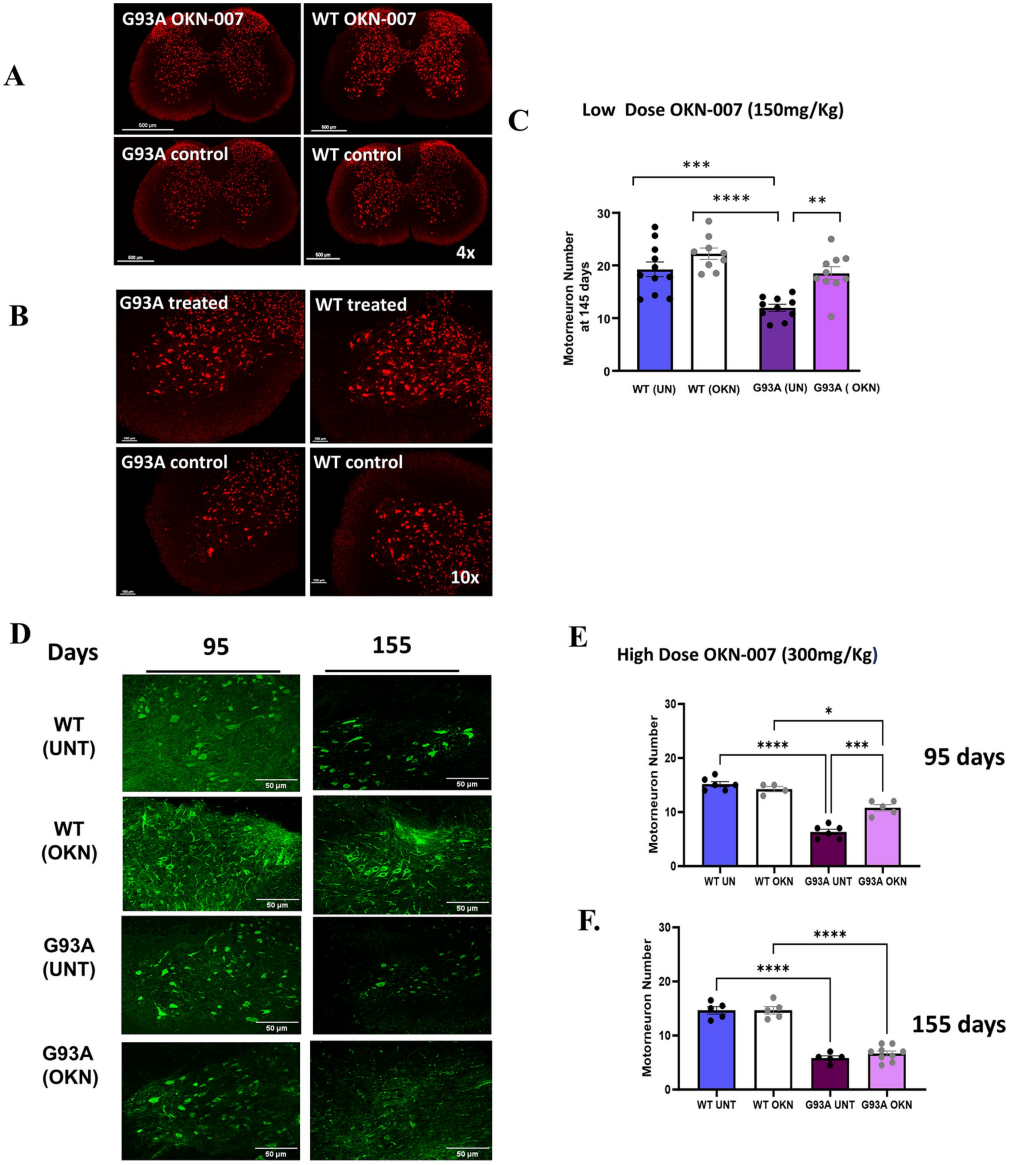
Neuroprotective effect of OKN-007: preservation of motor neurons in ALS mice. Mice were treated with a lower dose of OKN-007 (150 mg/Kg) beginning at 60 days of age and sacrificed at 145 days of age. **(A)** Shows a representative image of anti-NeuN immunostaining of a lumbar spinal cord cross-section including the ventral horn (4x) from treated and untreated WT and G93A mice stained with NeuN primary antibody to visualize *α*-MNs. **(B)** Shows a representative image of the ventral horn (10x) of treated and untreated WT and G93A mice used for α-MN counts. The quantification of neuronal staining of α-MNs in 5 sections of lumbar spinal cord in each mouse is shown in **(C)**. There is a significant α-MN loss (two-way ANOVA, genotype effect *p* < 0.0001, treatment effect *p* = 0.0002) in untreated G93A mice (*n* = 10) but not in the OKN-007 treated G93A group (*n* = 10) Tukey’s multiple comparisons *post hoc* test: G93A treated vs. G93A control *p* = 0.0016. Both treated WT (*n* = 9) and WT controls (*n* = 11) significantly differ from untreated G93A mice (Tukey’s *post hoc* test: WT control vs. G93A control *p* = 0.0003); Prism identified two outliers (Grubbs, alpha = 0.1), which were removed from the analysis; scorer was blinded to the group identity. Data are graphed as mean ± SEM. *denotes a significant difference between groups. There is a significant loss of α-MNs based on genotype (*p* < 0.0001) and a treatment effect (*p* = 0.0002) in untreated G93A mice (*n* = 10) but not in the OKN-007 treated G93A group (*n* = 10). In **(D)**, mice in Cohort 1 (*n* = 4–8 per group) were treated with a higher dose of OKN-007 (300 mg/Kg) beginning at approximately 25–40 days of age and sacrificed at 95 or 155 days of age. The panel shows a representative image of lumbar spinal cord ventral horn (20x) from treated and untreated WT and G93A mice stained with NeuN. The images and quantification of motor neuron number in **(E,F)** show a loss of motor neurons in the G93A spinal cord and a protection of α-MN number in G93A treated mice compared to untreated G93A mice (*p* < 0.001) at both 95 and 155 days. However, at 155 days of age, OKN-007 does not protect motor neuron loss in G93A mice. Data are expressed as mean ± SEM. * Denotes *p* < 0.05 determined using 2-way ANOVA with Tukey’s *post hoc* test (*n* = 5–6 per group).

To investigate the potential mechanisms underlying the delay in disease progression in OKN-007 treated G93A mice, we asked whether OKN-007 impacted α-MNs in the lumbar spinal cord region. Motor neuron counts in spinal cord sections revealed a higher number of α-MNs in the OKN-007 treated G93A mice (150 mg/kg) compared to the untreated controls at 145 days of age (Figures 3A–C). Control untreated G93A mice had an average of 11.98 ± 0.70 α-MNs per ventral horn, whereas the treated G93A mice exhibited an average of 18.22 ± 1.32 α-MNs. There is a significant α-MN loss (two-way ANOVA, genotype effect *p* < 0.0001, treatment effect *p* = 0.0002) in untreated G93A mice (*n* = 10, mean ± SEM = 11.98 ± 0.70) but not in the OKN-007 treated G93A group (*n* = 10, mean ± SEM = 18.22 ± 1.32; Tukeys multiple comparisons *post hoc* test: G93A treated vs. G93A control *p* = 0.0017). The α-MN numbers in the treated G93A mice were similar to those observed in WT mice (untreated WT: 19.27 ± 1.41, treated G93A: 18.22 ± 1.32). Treatment with the higher dose (300 mg/Kg) also significantly reduced motor neuron loss in the lumbar spinal cord region of G93A mice sacrificed at 95 days of age. 95-day old control G93A mice showed 6.3 ± 1.211 α-MNs while 10.8 ± 1.3 MNs were present in spinal cord sections from age-matched OKN-007 treated G93A mice (two-way ANOVA, *p* < 0.0001). No protection was found in response to OKN-007 treatment in 155-day old G93A mice where α-MN counts were 5.8 ± 0.4 in untreated versus 6.7 ± 0.5 in OKN treated mice (Figures 3D–F).

### Administration of OKN-007 does not alter astrocyte number in the lumbar spinal cord

**Figure 4:**
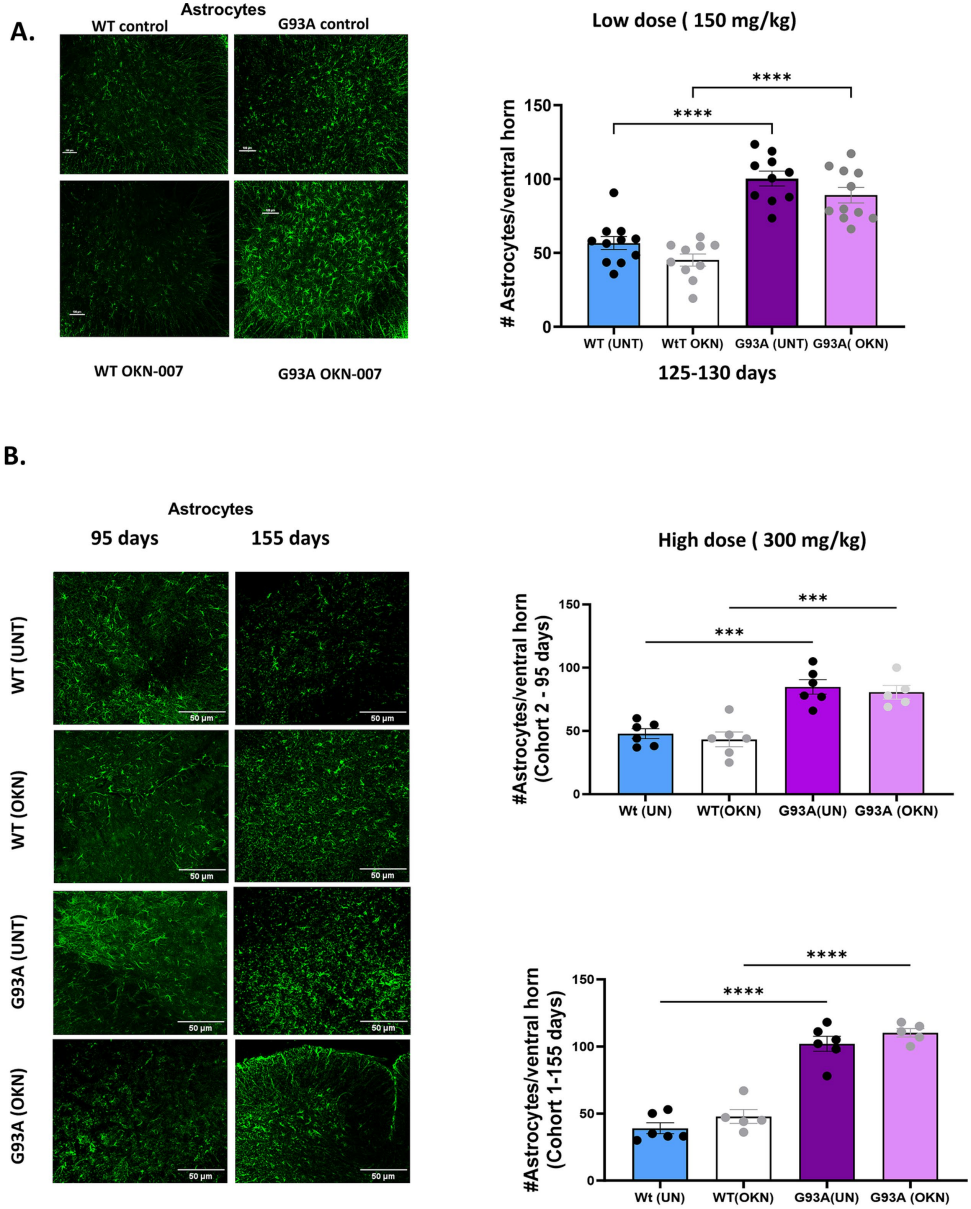
OKN-007 does not change astrocyte proliferation or activation in G93A mice. Representative images of lumbar spinal cord ventral horn sections from 125 to 130 day old wild-type control and G93A mice are shown in **(A)**, stained with a primary antibody for GFAP (green fluorescence) to visualize astrocytes after treatment with OKN at a dose of 150 mg/Kg that was initiated at 60 days of age in drinking water. The quantification at the right shows the number of astrocytes per ventral horn counted in 25 sections/mouse per group and averaged. The number of astrocytes is significantly higher in G93A mice and is not affected by treatment with OKN-007. Data were analyzed by 2-way ANOVA with Tukey *post hoc* multiple comparison test (*p* < 0.05). **(B)** Shows representative images of ventral horn in lumbar spinal cord stained with GFAP to visualize astrocytes in lumbar spinal cord from wildtype and G93A mice in Cohort 1 treated with 300 mg/kg OKN-007 from 30 to 45 days of age and sacrificed at either 95 (top) or 155 days of age (bottom) (*n* = 5–6 /group). Each data point represents average of number of GFP positive cells/mouse, using 2-way ANOVA with Tukey multiple comparison test (*p* < 0.05). Data are expressed as mean ± SEM. * denotes a significant difference. Similar to treatment with the lower dose of OKN-007, the number of astrocytes is increased in the G93A mice and not affected by OKN-007 at 95 or 155 days.

ALS has a strong inflammatory component that includes activation of astrocytes and microglia that can affect MN survival. Thus, we asked whether OKN-007 treatment altered the number of astrocytes in sections of the lumbar spinal cord. As shown in Figure 4A, the number of astrocytes in sections of lumbar spinal cord, identified using GFAP staining, was nearly doubled in spinal cord of the G93A mice at 125–130 days of age. This was not altered in response to treatment with 150 mg/kg of OKN-007 initiated at 60 days of age. Figure 4B shows a similar response to the higher dose treatment in Cohort 1 (treated from 30–45 to 155 days of age) and Cohort 2 (treated from 30–45 to 95 days). We compared the astrocyte number in WT and G93A mice control groups and those treated with OKN. The astrocyte numbers are approximately doubled in the G93A mice, even in the mice in Cohort 2 that are only 95 days old. However, our results do not show a significant effect of OKN-007 treatment in either WT or G93A mice.

### Administration of OKN-007 does not impact the microglia reactivity in the spinal cord of G93A mice

**Figure 5:**
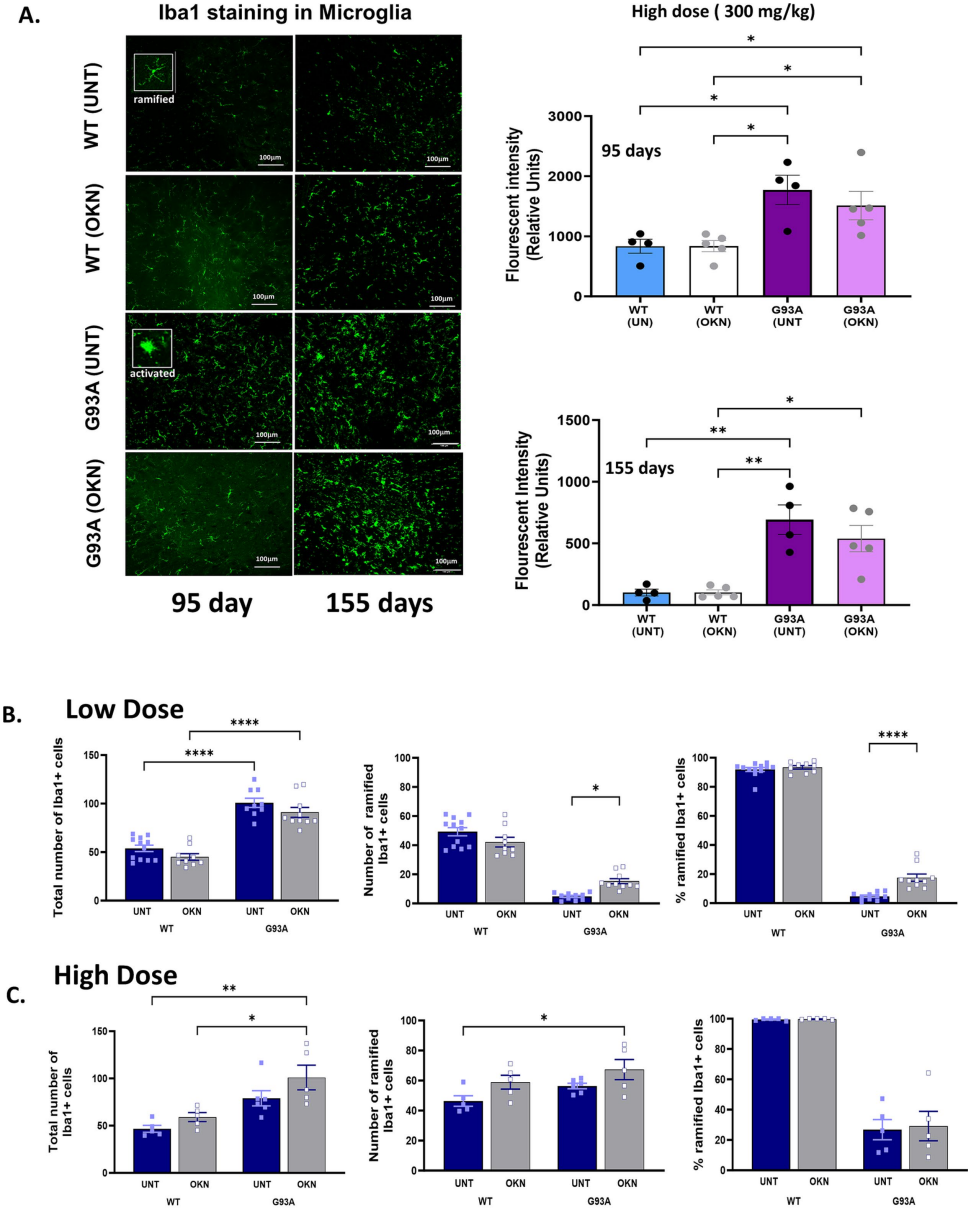
Impact of OKN-007 treatment on microglia number and activation in the lumbar spinal cord of G93A mice. The effect of high dose treatment (Cohort 1) on microglia in lumbar spinal cord ventral horn is shown in **(A)**. Representative images and quantification of Iba1 staining in the ventral horn of the lumbar spinal cord in both WT and G93A mice are shown in response to treatment with OKN-007 for a duration of 95 or 155 days. Data were quantified and analyzed using 2-way ANOVA. Data are expressed as mean ± SEM. * denotes a significant difference with *p* < 0.05. **(B,C)** Show the quantification of the total number of microglia and the percentage of ramified in lumbar spinal cord ventral horn sections in WT and G93A mice in response to low and high dose OKN-007 treatment. In **(B)**, mice were treated with 150 mg/kg OKN-007 in drinking water initiated at 60 days of age and sacrificed at from 145 days. The total number of microglia in the ventral horn of lumbar spinal cord sections was determined using Iba1 staining. There is a strong genotype effect (two-way ANOVA, *p* < 0.0001) on microglia proliferation (treatment effect *p* = 0.0547, no significant difference in Tukey’s *post hoc* between treated and untreated G93A mice). In addition, the number and percentage of ramified (surveillant) microglia versus highly rounded amoeboid activated microglia were determined in 5 sections per mouse from 5 mice per group. Assessment of ramified versus activated microglia (both number and percentage of ramified microglia) in response to the low dose of OKN reveals a significant loss in ramified microglia indicating increased activated microglia (*p* < 0.0001) in G93A mice compared to control wild-type animals that is reduced by OKN-007 treatment (*p* < 0.022) compared to the control vehicle-treated G93A group. The number of non-activated, ramified microglia per ventral horn shown in the middle panel reveals a strong genotype effect (two-way ANOVA, *p* < 0.0001) and interaction (*p* = 0.0008). Low dose OKN-007 decreases microglial activation in G93A mice (Tukey’s *post hoc* test for treated G93A vs. untreated *p* = 0.0212). Data are expressed as mean +/− SEM; *n* = 9–12 sections per group. Data were analyzed using 2-way ANOVA with Tukey’s *post hoc* test. A similar trend is seen for the percentage of ramified cells. Non-activated, ramified microglia count expressed as a percentage of all Iba1-positive cells per ventral horn shows a strong genotype effect (two-way ANOVA, *p* < 0.0001), treatment effect (*p* < 0.0001), as well as interaction (*p* = 0.0012). **(C)** Shows the effect of high dose treatment (Cohort 1) on microglia content and ramified microglia in lumbar spinal cord ventral horn. Representative images and quantification of Iba1 staining in the ventral horn of the lumbar spinal cord in both WT and G93A mice in response to treatment with OKN-007 for a duration of 95 or 155 days. Data were quantified and analyzed from 20 to 25 sections using 2-way ANOVA with Tukey’s *post hoc* test and are expressed as mean ± SEM. * Denotes a significant difference with *p* < 0.05.

Microglia are specialized macrophages that are crucial for maintaining homeostasis in the CNS (Leyh et al., 2021). To investigate the impact of OKN-007 on microglial reactivity, we measured microglia as Iba1^+^ cells in the lumbar region of the spinal cord. Shown in Figure 5A is the fluorescence intensity of Iba1 stained cells in lumbar spinal cord sections from the high dose experiment at 95 or 155 days of age. Interestingly, Iba1 fluorescence intensity increased by 89% in the 95-day old untreated G93A group (two-way ANOVA, *p* = 0.021). Iba1 fluorescence also increased (~76%) in the 95-day old OKN-007 treated G93A group. By 155 days of age, Iba1 fluorescence intensity was elevated 5 to 7-fold in both untreated and OKN-007 treated G93A mice. We also determined the number of both reactive (amoeboid) and homeostatic (ramified) microglia based on their morphology as illustrated in the insets in Figure 5A. Figures 5B,C show the total number of Iba1 positive cells as well as the number and percentage of homeostatic (ramified) cells in response to the low (Figure 5B) and high dose (Figure 5C) OKN-007 treatment at 145 or 155 days, respectively. The total number of microglia significantly increased (approximately doubled) in G93A animals compared to control animals and was not affected by either high or low dose OKN-007 treatment. In response to the low dose treatment from 60 to 145 days, OKN-007 significantly decreased microglia reactivity in G93A mice, resulting in an increase in the number and percentage of homeostatic, ramified microglia per ventral horn. In contrast, a higher dose of OKN-007 administered from 30–45 days to 155 days did not result in any alteration in the number or ramification of microglia (Figure 5C).

### Lower dose OKN treatment reduced NMJ fragmentation

**Figure 6:**
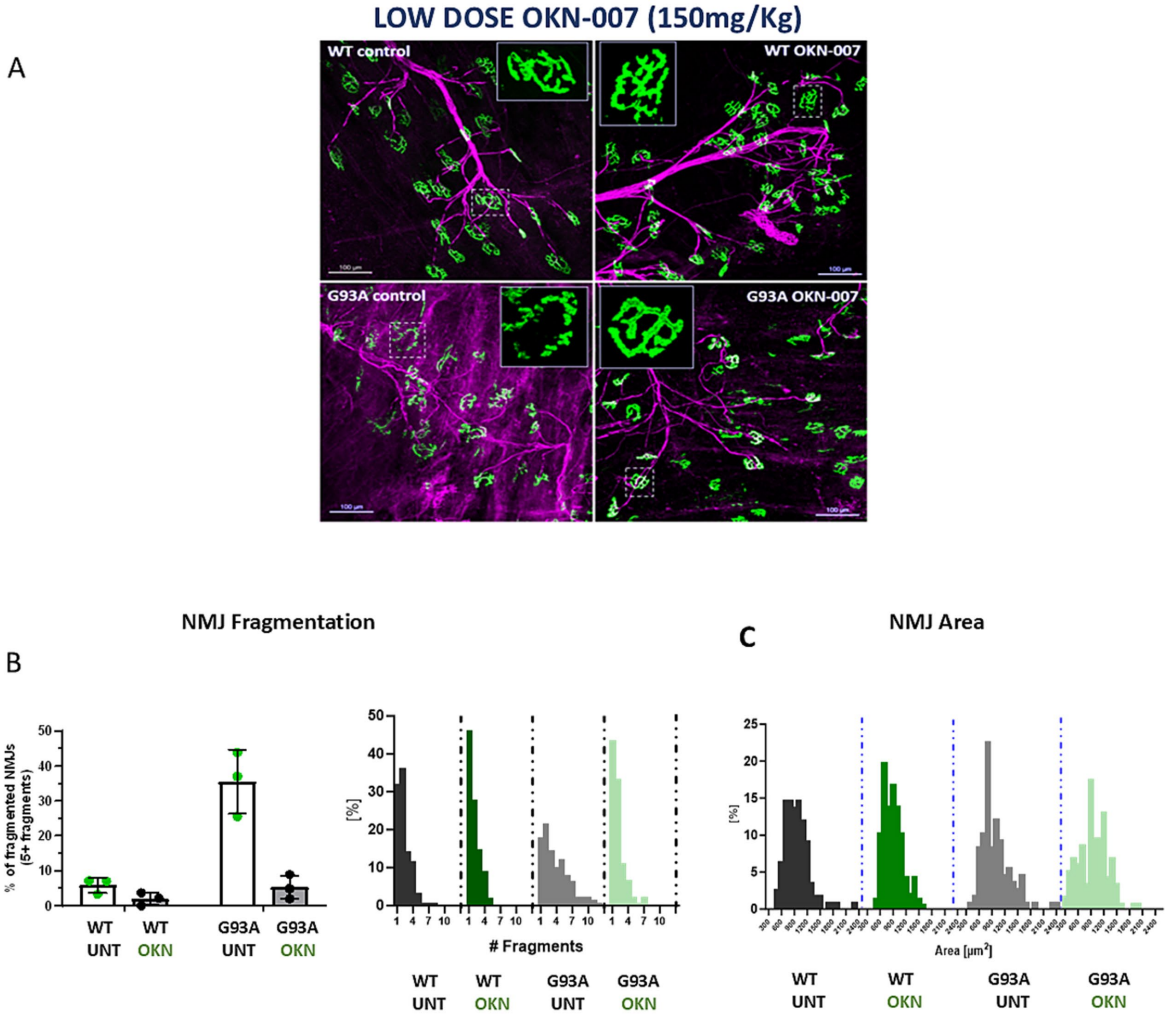
Representative image of the neuromuscular junctions (NMJs) (20x) from treated and untreated WT and G93A female mice (*n* = 3/group). Four muscles per group were analyzed and approximately 100 endplates were evaluated for each muscle **(A)**. The NMJs were immunostained for SV2 and 2H3 (pre-synaptic component; in magenta) and labeled with bungarotoxin conjugated to a fluorophore (post-synaptic component– AchR; in green), including a close-up view of a single acetylcholine receptor (AChR) in the inset. **(B)** Shows a graphic representation of the percentage of NMJ fragmentation. A significant reduction in NMJ fragmentation is observed in the G93A treated group compared to untreated G93A controls (Tukey’s *post hoc* test *p* = 0.0004; two-way ANOVA, genotype effect *p* = 0.0005, treatment effect *p* = 0.0004). the right panel shows the distribution of NMJ fragment number, with a clear right-shift in G93A control mice. In **(C)**, quantification of NMJ area shows no significant difference between treated G93A and untreated G93A mice.

Because we observed an increase in motor neuron survival in OKN-007 treated G93A mice, we asked whether there was any protective effect at the level of the neuromuscular junction (NMJ). There is a clear preservation of NMJ morphology as illustrated in the inset of single acetylcholine receptors in Figure 6A. Despite the changes in morphology, we found no difference in NMJ area in treated or untreated G93A mice at 145 days of age. However, there is a highly significant effect of OKN-007 on NMJ (AChR) fragmentation as determined by counting the number of fragmented pieces of each AChR cluster. On average 35% of NMJs in untreated G93A mice are fragmented, while the treatment with OKN-007 decreases NMJ fragmentation to 5% (two-way ANOVA, Tukey’s *post hoc* test *p* = 0.0004; Figure 6B, left panel). There is also a clear difference in the distribution of the number of NMJ fragments between untreated G93A mice and the remaining groups (Figure 6B, right panel).

### OKN-007 treatment restores lumbar spinal cord perfusion levels in G93A mice and increases levels of myo-inositol

**Figure 7:**
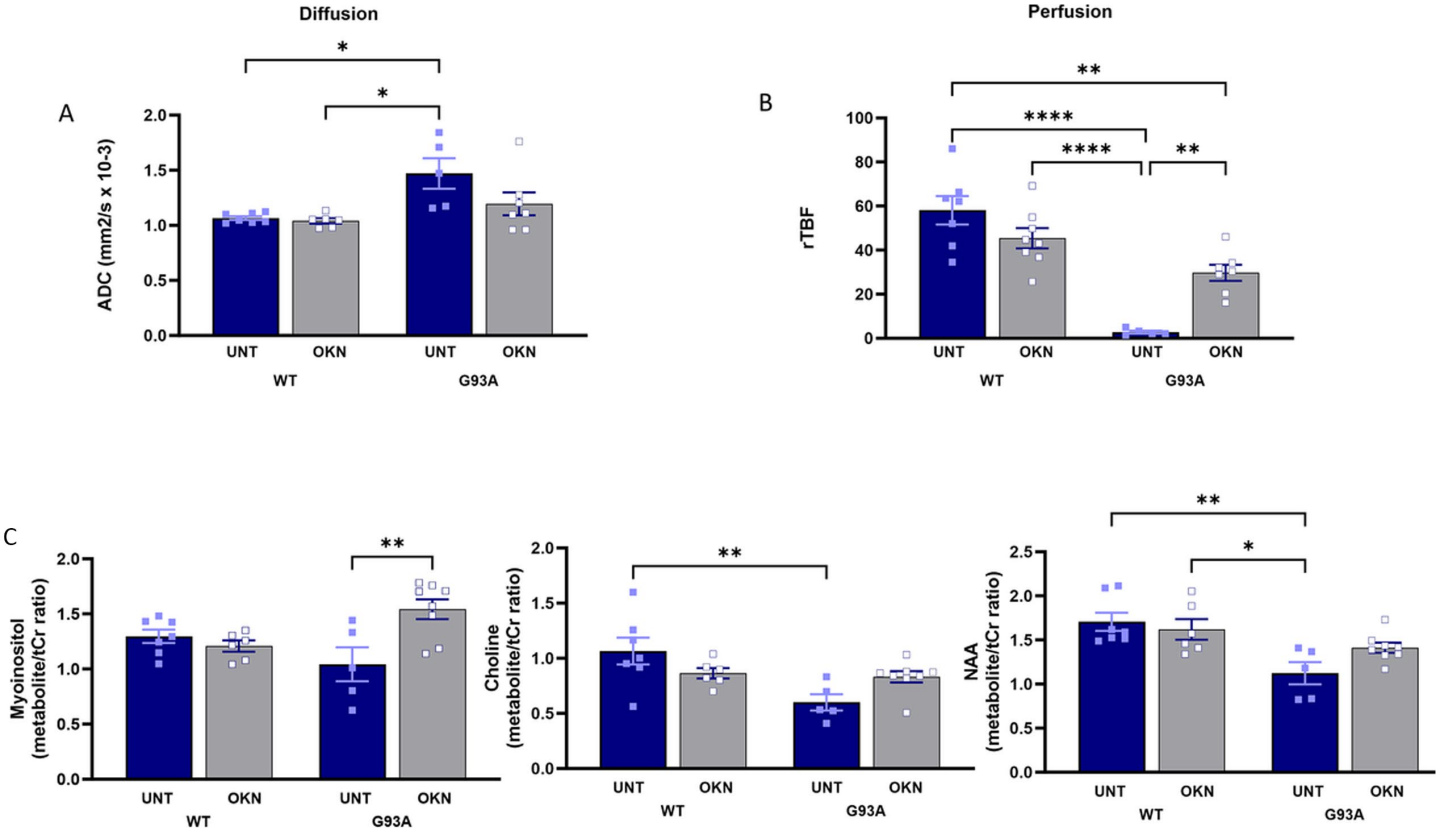
OKN 007 low dose treatment normalizes perfusion and diffusion in the lumbar spinal cord and affects the levels of myo-inositol. **(A)** Illustrates the measurement of **(A)** diffusivity (apparent diffusion coefficient, ADC) and in **(B)** the perfusion (relative tissue blood flow, rTBF) using MRI analysis is shown in mice treated with 150 mg/kg and untreated control animals. (*n* = 5–6 mice per group) **(C)**. Myo-inositol, choline and N-acetyl aspartate (NAA) metabolite levels were measured using MRS in the spinal cord of animals treated with 150 mg/kg and untreated animals. The results are presented as mean ± SEM, with a sample size of *n* = 5–8 per group.

Amyotrophic lateral sclerosis (ALS) disrupts tissue structure in the lumbar spinal cord of control G93A mice, evidenced by a higher apparent diffusion coefficient (ADC) that is measured through diffusion-weighted MRI (DWI). As shown in Figure 7A, we found a 37% increase in ADC in untreated G93A mice at 145 days of age. Mice treated with 150 mg/kg OKN-007 from 60 to 145 days of age also showed an increase in ADC, but this was not statistically significant by two-way ANOVA, suggesting the OKN-007 may have tempered the increase. Results from the perfusion MRI analysis (Figure 7B) showed a significant reduction in relative tissue blood flow (rTBF) in the spinal cord of untreated G93A mice compared to WT mice, supporting impaired vascularization or functional deficit. Notably, OKN-007 treatment significantly increased blood flow in G93A mice (interaction effect *p* = 0.0004, Tukey’s *post hoc* test G93A treated vs. untreated *p* = 0.0057). Despite the substantial difference in perfusion rates between treated and untreated G93A mice, there was no significant difference in blood vessel density, as indicated by anti-CD31 immunostaining (data not shown). Additionally, MR spectroscopy data (Figure 7C) revealed lower myo-inositol levels in control G93A mice, indicative of neuroinflammation, that is reversed by OKN-007 treatment (two-way ANOVA, treatment effect *p* = 0.0351, interaction *p* = 0.0040, Tukey’s *post hoc* test G93A treated vs. untreated *p* = 0.0049).

### Differential effects of shorter- and longer-term treatment with OKN-007 (300 mg/kg) on levels of inflammatory cytokines in the spinal cord

**Figure 8:**
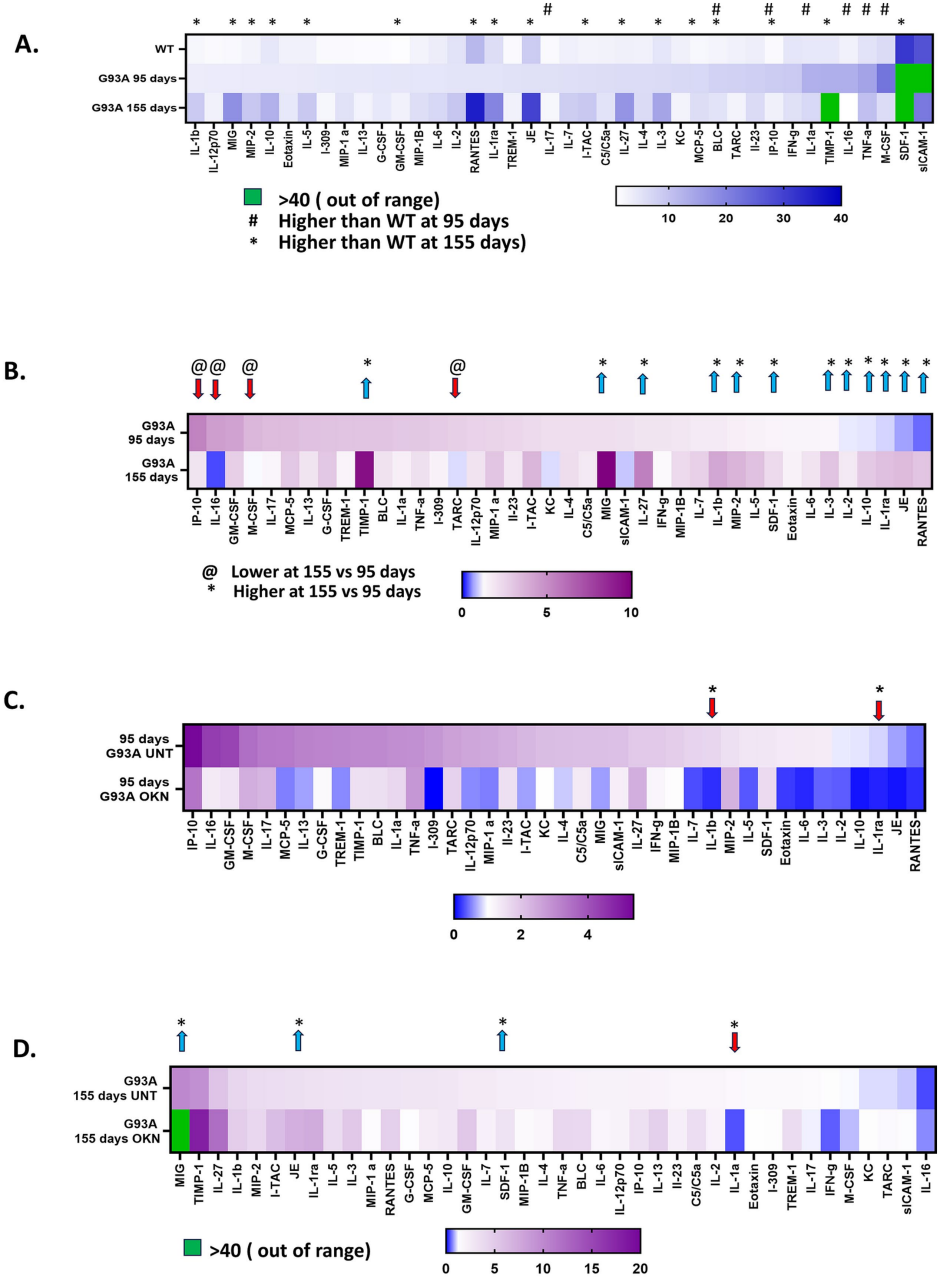
Effect of OKN-007 on the expression of inflammatory cytokines/chemokines in the spinal cord of WT and G93A mutant mice. A cytokine/chemokine protein array (Mouse Cytokine Array **(A)** #ARY006, R&D Systems, Minneapolis, MN) was used to detect the levels of 40 proteins related to inflammation in spinal cord extracts from WT and G93A mice at 95 and 155 days of age after treatment with OKN-007 at 300 mg /kg or vehicle in drinking water (*n* = 6 mice per group). **(A)** Shows the mean absolute values ± SEM of protein levels expressed in spinal cord extracts from wildtype and G93A untreated mice (*n* = 6 mice per group). Seven out of the 40 proteins measured were significantly higher in G93A mice at 95 days (denoted by #) and 17 were different at 155 days (denoted by *) as determined using multiple unpaired *t*-tests. **(B)** Shows a comparison of the expression of the 40 cytokines in the spinal cord from untreated G93A mice as a function of disease progression (*n* = 6) sorted by the highest expression in G93A mice at 95 days. Values are shown (mean ± SEM) for samples from untreated G93A mice expressed relative to untreated WT values at 95 and 155 days of age. Of the 40 proteins analyzed, 16 are significantly different at 155 days compared to 95 days in spinal cords from G93A mice as determined using multiple unpaired *t* tests. Twelve are increased by 95 days compared to wildtype values (*) and 4 are lower at 95 days (@). Heatmaps in **(C,D)** show a comparison of the levels of cytokine and chemokine proteins in G93A mice at 95 days **(C)** and 155 days **(D)** after treatment with OKN-007, sorted by the highest expression in G93A mice at 95 days in **(C)** and sorted by highest expression in G93A at 155 days in **(D)**. * Indicates a significant difference between groups. The data were analyzed using multiple unpaired *t*-tests for each cytokine setting significance at *p* < 0.05. The results are presented as mean ± SEM, with a sample size of *n* = 6 per group.

To investigate the impact of OKN-007 treatment on inflammation in the spinal cord of G93A mice, we performed quantitative analysis of proinflammatory cytokines and chemokines in spinal cord extracts using a protein array. The heat map in Figure 8A shows the effect of the G93A transgene on cytokine expression in spinal cord during ALS disease progression. Mean absolute values are represented for WT untreated mice (combining mice at 95 and 155 days) and untreated G93A mice at 95 or 155 days. Seven out of the 40 proteins measured were elevated early in disease at 95 days, including BLC, IL-1α, IL-16, IL-17, IP-10, M-CSF and TNFα. By 155 days into disease progression, the expression of 17 cytokine related proteins was different than WT. Figure 8B shows a comparison of the expression of 40 cytokines in the spinal cord from untreated G93A mice as a function of disease progression change between 95 and 155 days. Normalized values are shown (mean ± SEM) for samples from untreated G93A mice relative to untreated WT values at 95 and 155 days of age. Of the 40 proteins analyzed, 16 were significantly different at 155 days compared to 95 days in spinal cords from untreated G93A mice. IL-1β IL-1ra, IL-27, JE/MCP-1, MIG, RANTES and TIMP showed the highest fold elevation during disease progression. Notably, while most cytokines that showed a significant change were increased at 155 days relative to 95 days, four proteins (IL-16, IP-10, M-CSF and TARC) showed lower expression at 155 days than 95 days. In Figures 8C,D, the effect of OKN-007 treatment on cytokine expression is shown for spinal cord from 95 (**C**) and 155-day (**D**) G93A mice. At 95 days of age, OKN treatment significantly reduced expression of IL-1β and IL-1ra. After 155 days, IL-1α protein levels were reduced by OKN treatment and three proteins (JE/MCP-1, MIG and SDF-1) were significantly elevated in OKN-007 treated G93A mice.

### OKN-007 had limited effect on mRNA transcript levels in the spinal cord of WT and G93A mice

**Figure 9:**
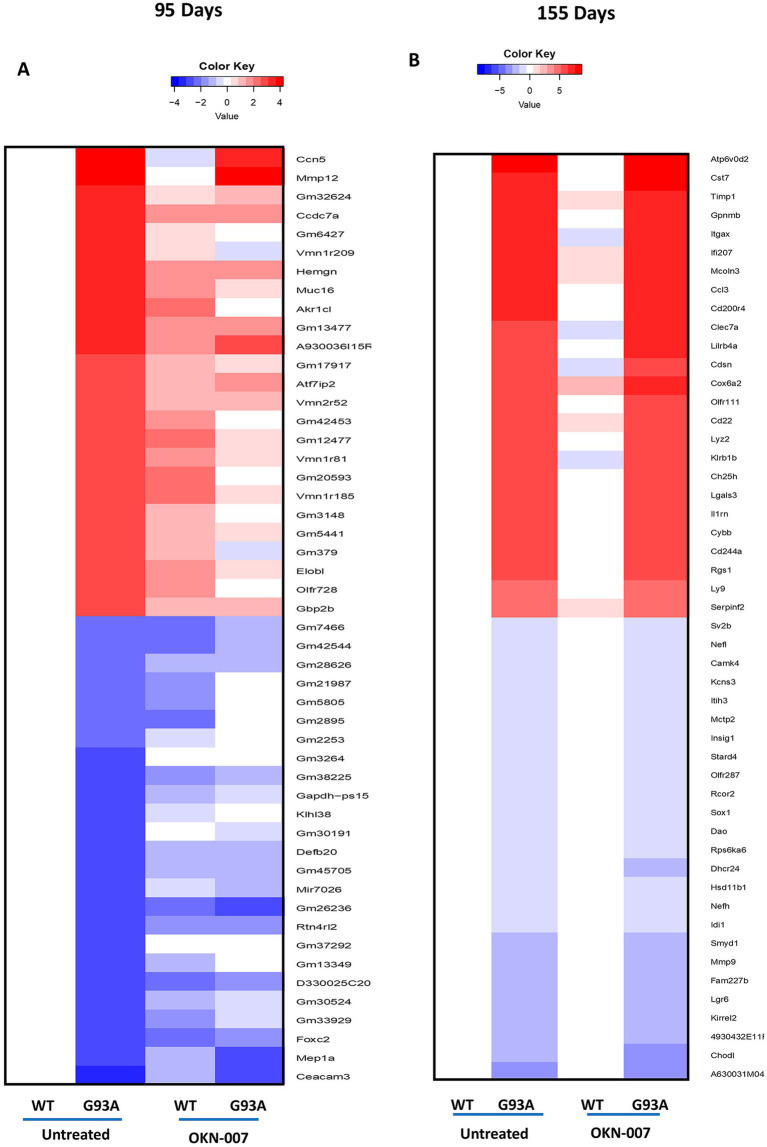
RNA seq analysis. Heatmaps depicting the mRNA expression levels of genes in spinal cord from wildtype and G93A mice, untreated and with OKN-007 treatment. Values shown are differences relative to values in wildtype untreated mice, at day 95 **(A)** and 155 **(B)**, respectively. Genes listed are the 25 most upregulated and downregulated, ranked by log-fold change, when comparing wildtype and G93A mice without OKN-007 treatment. Since untreated wildtype is the base value for comparison, its column is blank.

Using RNA sequencing analysis (RNA-seq), we found significant changes in expression between WT and G93A mice as expected. While there was evidence for trends of potential change in either expression magnitude or direction after treatment with OKN-007, few differences reached the level of statistical significance (Figure 9; Supplementary Figure S4). These trends were most evident at 95 days (Figure 9A). Overall, these findings highlight the need for additional investigation into the potential therapeutic effect of OKN treatment in reversing the dysregulation of these genes associated with the G93A model.

### OKN-007 does not affect muscle mass, force generation, and mitochondrial respiration or lifespan in G93A mice

To assess muscle mass, we measured muscle wet weights of gastrocnemius muscle at 95, 135, and 155 days in wildtype and G93A transgenic mice that were either treated with OKN-007 or untreated. Muscle mass was reduced by ~50% at all time points in G93A mice when compared to wildtype mice, and treatment with OKN-007 had no effect on gastrocnemius muscle mass in both wildtype and G93A transgenic mice (Supplementary Figure S2A). We also measured muscle force generation in EDL muscle. Both maximal and specific force generation were ~ 50% lower in G93A transgenic mice when compared to wildtype mice, while treatment with OKN-007 had no effect on muscle force generation (Supplementary Figure S2B).

In addition to muscle mass and force generation, we measured mitochondrial respiration and hydroperoxide generation in permeabilized fiber bundles from gastrocnemius muscle. At 95 days, basal hydroperoxide generation was not different between groups (Supplementary Figure S3A); however, maximally stimulated complex I + II mitochondrial respiration was significantly lower in G93A transgenic mice when compared to wildtype mice with no effect from OKN-007 treatment (Supplementary Figure S3B). At 155 days, basal hydroperoxide generation was significantly elevated in G93A mice when compared to wildtype mice, and treatment with OKN-007 further increased hydroperoxide generation on G93A transgenic mice when compared to untreated G93A transgenic mice (Supplementary Figure S3C). At 155 days, mitochondrial respiration was not different between groups (Supplementary Figure S3D).

To test whether OKN -007 treatment can alter the survival of the G93A mice, we measured lifespan in a cohort of untreated G93A female mice (*n* = 21) and a cohort of G93A mice treated with 300 mg/kg OKN-007 (*n* = 17) initiated at approximately 30 to 45 days of age. OKN-007 did not alter lifespan of the G93A mice (Supplementary Figure S1).

## Discussion

Our study aimed to investigate the potential therapeutic effects of OKN-007 on the progression of amyotrophic lateral sclerosis (ALS) using the SOD1 G93A mutant mouse model. OKN-007 is a nitrone-based compound known for its antioxidant, anti-inflammatory, and neuroprotective properties (Floyd et al., 2011; Clausen et al., 2008; Floyd et al., 2008; Culot et al., 2009; Kuroda et al., 1999). We found that OKN-007 had positive effects on the ALS phenotype, especially in the early stages of the disease. For example, OKN-007 treatment initiated before disease onset was neuroprotective and reduced the loss of *α*-MNs near disease onset and preserved neuromuscular junction integrity in later stage disease. OKN-007 also dampened microglial activation, elevated perfusion in spinal cord and increased levels of myo-inositol, consistent with reduced neuroinflammation. Consistent with this, the expression of several proteins associated with inflammation is increased in spinal cord extracts from G93A mice and OKN-007 dampened the expression of IL-1β and IL-1ra at onset and IL-1*α* at late-stage disease.

A promising result of our study is the delay in the progression of ALS symptoms in G93A mice treated with OKN-007. Both the lower (150 mg/kg) and higher (300 mg/kg) doses of OKN-007 significantly slowed disease progression, particularly at earlier stages, i.e., before mice showed signs of paralysis. While the treatment did not significantly delay the initial onset of symptoms (score 1), it effectively postponed the advancement to more severe stages of ALS symptomatology (scores 2 and 3). We found that treated mice had a higher median age before they reached scores 2 when compared to untreated controls. It is also interesting to note that the lifespan of the mice was not significantly altered regardless of the dose used and surprisingly, the untreated controls tended to live longer despite reaching advanced ALS symptoms at a younger age. More work is needed to understand why advanced ALS symptoms are delayed with OKN-007 treatment, but lifespan is not altered.

One contributing factor to the delayed progression of ALS is likely the dramatic effect of OKN-007 on preserving the α-MN number. Motor neuron counts in the lumbar spinal cord were notably higher in OKN-007 treated G93A mice, underscoring the potential of OKN-007 to protect motor neurons from degeneration. This protective effect mimics our previous results showing that OKN-007 can preserve motor neuron counts in old mice (Piekarz et al., 2022). It is interesting to note that the antioxidant Edaravone, a current ALS therapy, was also able to slow motor neuron loss and symptom progression in G93A mice (Ito et al., 2008) and to reduce motor neuron loss in Wobbler mice, a model of neurodegeneration. The neuroprotection afforded by OKN-007 treatment in the G93A mice could be a direct effect leading to preservation of motor neurons or could be indirectly elicited through the effect of OKN-007 on non-neuronal cells, i.e., astrocytes, microglia and other glial cell types.

Indeed, while damage to motor neurons leads to disease onset and affects disease progression and survival, previous studies have shown that ALS is not motor–neuron autonomous and that glial cells play important roles in motor neuron degeneration. Specifically, expression of mutant SOD1 in motor neurons alone does not lead to an ALS phenotype (Pramatarova et al., 2001) supporting the importance of non-neuronal cells. In addition, reduced expression of mutant SOD1 in motor neurons delays disease onset and slows early disease progression but does not affect later stage disease progression or survival (Boillée et al., 2006; Yamanaka et al., 2008). Activation of both astrocytes and microglia are established pathological hallmarks of neuroinflammation in ALS (Hall et al., 1998). Activated microglia in G93A mice have been shown to produce TNF-α, IL-1β, and superoxide (Zhao et al., 2010) that can induce muscle denervation, atrophy, and motor neuron cell death (Komine and Yamanaka, 2015).

To address the potential anti-inflammatory impact of OKN-007 on microglia and astrocytes in the G93A transgenic mice, we measured activation of microglia and astrocytes. Treatment with OKN-007 reduced microglial activation at the lower dose, as evidenced by the increased number of non-activated, ramified microglia. This anti-inflammatory effect on microglia was not observed at the higher dose or in longer treatment durations. It appears that the benefits of OKN-007 on microglial activation may be dose dependent and may diminish over time with disease progression. Interestingly, G93A mice showed nearly double the astrocyte numbers in the lumbar spinal cord compared to wild-type mice and OKN-007 treatment did not significantly alter astrocyte numbers. This indicates that while OKN-007 may modulate microglial activity, its impact on astrocyte proliferation or activation may be limited. Since astrocytes maintain low extracellular concentrations of glutamate, the lack of effect of OKN-007 on astrocytes suggests that OKN-007 may not be effective in reducing the excitotoxicity associated with ALS.

ASL MRI revealed that OKN-007 treatment improved lumbar spinal cord perfusion (rTBF) in G93A mice, indicating enhanced blood flow. Previously, reported OKN007 restores the LPS induced blood brain barrier in rats compared with its vehicle treated controls (Towner et al., 2019a). Furthermore, MR spectroscopy showed increased levels of myo-inositol, a marker of neuroinflammation, in untreated G93A mice, which was normalized by OKN-007 treatment. This supports the anti-inflammatory properties of OKN-007 and its role in reducing neuroinflammation in ALS.

ALS is accompanied by progressive paralysis in hindlimbs that is followed by loss of innervation (McIntosh et al., 2023; Alhindi et al., 2022). This is first evident in the SOD1G93A mouse at P14-P30 for fast-twitch muscles in asymptomatic G93A mice and by P30, 20% of fibers in the gastrocnemius and up to 40% of fibers in the tibialis anterior are already denervated (Gould et al., 2006; Vinsant et al., 2013). Here we measured NMJ morphology, fragmentation and area in WT and G93A transgenic mice at 145 days of age. OKN-007 (150 mg/kg bwt) appears to partially protect the NMJ as OKN-007 treated G93A mice had a lower percentage of fragmented NMJs. However, we did not observe a significant effect of OKN-007 on overall muscle mass (Supplementary Figure S2), suggesting that while NMJ preservation is beneficial, it may not be sufficient alone to prevent muscle atrophy in ALS.

Cytokines mediate the immune response and control cell proliferation, differentiation and cell survival/apoptosis, and are involved in several pathophysiological processes. Indeed, elevated levels of pro-inflammatory cytokines and neuroinflammatory processes are implicated in the initiation and progression of amyotrophic lateral sclerosis (ALS) (Nguyen et al., 2004). In agreement with previous studies, we found increased expression of inflammatory cytokines and chemokines in spinal cord from G93A mice. Seven of the 40 proteins measured by the cytokine protein panel used in this study were elevated in G93A spinal cord near onset (at 95 days) specifically, BLC/CXCL13, GM-CSF, IL-1*α*, IL-16, IL-17, IP-10/CXCL10, M-CSF, and TNFα and by 155 days 17 inflammatory proteins were elevated relative to levels measured in wildtype mice, including IL-1β and IL-1Ra, the anti-inflammatory cytokine IL-10 and several cytokines involved in attraction and activation of monocytes (TIMP-1, JE/MCP-1), neutrophils (MIP2), T cells (MIG/CXCL9, RANTES, TARC) and lymphocytes (SDF-1/CXCL12). A number of previous reports have noted elevated levels of proinflammatory cytokines in spinal cord of G93A mice, and in particular, several reports show increased levels of IL-1β, IL-α and IL-1Ra (Hensley et al., 2002; Hensley et al., 2003; Kiaei et al., 2006; Kim et al., 2006; Kassa et al., 2009; Neymotin et al., 2009). Notably, at 95 days of age, OKN-007 treatment significantly reduced expression of IL-1β and IL-1Ra and by 155 days, IL-1α protein levels were also reduced by OKN treatment. IL-1β has been suggested as an initiating signal of neuroinflammation in ALS (Meissner et al., 2010). It is produced by macrophages and is a member of the interleukin-1 cytokine family. IL-1β levels have been shown to be elevated in the CNS of mutant SOD1 transgenic mice and in the cerebrospinal fluid of ALS patients (Li et al., 2000). Preclinical studies show that blocking IL-1 can slow the progression of ALS. For example, neutralizing IL-1b with antibody treatment was shown to reduce neuroinflammation and improve ALS phenotypes in an ALS mouse model (Hu et al., 2023) IL-1RA modulates a variety of interleukin 1 related immune and inflammatory responses and is an endogenous inhibitor of the pro-inflammatory effect of IL1β and Il-1alpha that acts as a competitive inhibitor of the IL-1 receptor (Perrier et al., 2006). IL-1α is produced by monocytes and macrophages and released in response to cell injury inducing apoptosis and activation of NF-kB and the p38, p42/p44 and JNK pathways (Wu et al., 2004). Thus, OKN-007 may modulate inflammation through indirect dampening of these proinflammatory signals. Interestingly, at 155 days, three cytokines (JE/MCP-1, MIG/CXCL9 and SDF-1/CXCL12) were significantly elevated in OKN-007 treated G93A mice. As mentioned above, these cytokines are involved in attracting immune cells. Continued activation of these proteins by OKN-007 may be the result of a continued attempt to resolve neuroinflammation and neuropathology. Together these results indicate that OKN-007 treatment reduced the expression of proinflammatory molecules in the spinal cord in early-stage disease, suggesting its potential to alleviate inflammation and delay disease progression by protecting the motor neurons. However, while OKN-007 may initially delay disease progression by safeguarding the motoneurons, its effectiveness might diminish in end stage disease. The ability of OKN-007 to normalize the expression of these genes highlights its potential to correct disease-related molecular alterations.

In conclusion, our study demonstrates that OKN-007 has promising molecular qualities that make it a good therapeutic candidate. By delaying disease progression, preserving motor neurons, reducing neuroinflammation, and protecting NMJ integrity, OKN-007 shows promise as a disease-modifying treatment for ALS. However, its effects on muscle mass and astrocyte activation appear limited, indicating the need for combination therapies or additional strategies to address these aspects of ALS pathology fully. Future studies should explore the optimal dosing regimens and the long-term effects of OKN-007 to fully elucidate its therapeutic potential and mechanisms of action in ALS.

## Data Availability

The data presented in the study are deposited in the GEO repository, accession number GSE281064.

## Supplementary material

SUPPLEMENTARY FIGURE S1 Survival. OKN-007 does not improve survival in G93A mutant mice. Graph shows survival for control untreated mice (*n* = 21, hatched line) or mice treated with 300mg/kg body weight OKN-007 in drinking water beginning at 30–45 days of age (*n* = 17, solid grey line). Data were analyzed using the Mantel-Cox log rank test.

SUPPLEMENTARY FIGURE S2 Effect of OKN 007 in skeletal muscle of G93A mice. **(A)** Gastrocnemius muscle mass, normalized to body weight in response to a low dose of OKN007 (150 mg/kg) at 135 days or high dose (300mg/kg) at 95 and 155 days of age. **(B)** Assessment of muscle contractile function in G93A animals after treatment with OKN at a dosage of 300 mg/kg body weight for both 95 and 155 days. All values are presented as mean ± SEM, with a sample size of *n* = 10–12 mice per group.

SUPPLEMENTARY FIGURE S3 Effect of OKN 007 in gastrocnemius muscle mitochondrial respiration of G93A mice. **(A)** State 1 Hydroperoxide generation in permeabilized fiber bundles from red gastrocnemius muscle in 95-day old WT and G93A mice. **(B)** Maximally stimulated complex I + II mitochondrial respiration in permeabilized fiber bundles from red gastrocnemius muscle in 95-day old wildtype and G93A mice. **(C)** State 1 Hydroperoxide generation in permeabilized fiber bundles from red gastrocnemius muscle in 155-day old WT and G93A mice. Panel D. Maximally stimulated complex I + II mitochondrial respiration in permeabilized fiber bundles from red gastrocnemius muscle in 155-day old WT and G93A mice. All values are presented as mean ± SEM, with a sample size of *n* = 3–7 mice per group.

SUPPLEMENTARY FIGURE S4 RNA seq analysis. Heatmaps depicting the mRNA expression levels of genes in spinal cord from wildtype and G93A mice, untreated and with OKN-007 treatment. Values shown are log2fold differences relative to values in wildtype untreated mice, at day 95 **(A)** and 155 **(B)**, for genes differentially expressed with an uncorrected *p*-value cutoff of <0.01 when comparing the untreated G93A group to the untreated wildtype group. Because untreated wildtype is the base value for comparison, its column is blank.
