# Supplementary material for: The nitrone compound OKN-007 delays motor neuron loss and disease progression in the G93A mouse model of amyotrophic lateral sclerosis
Source: Front Neurosci. 2024 Nov 20;18:1505369. doi: 10.3389/fnins.2024.1505369 (PMC11614777; doi:10.3389/fnins.2024.1505369)
Supplement: Supplementary file 1 [file Presentation_1.pptx]

## Slide 1
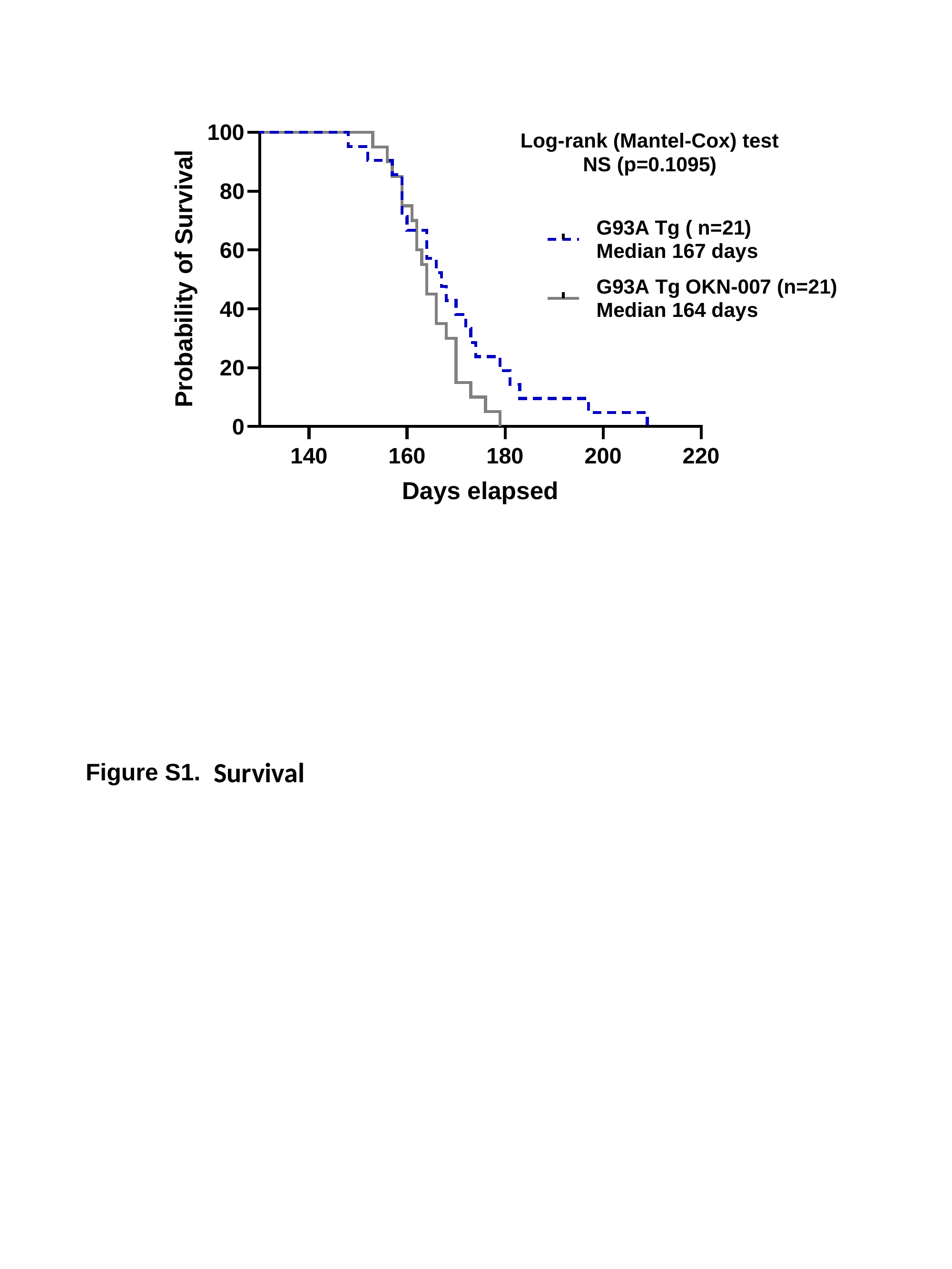

Survival
Figure S1.

## Slide 2
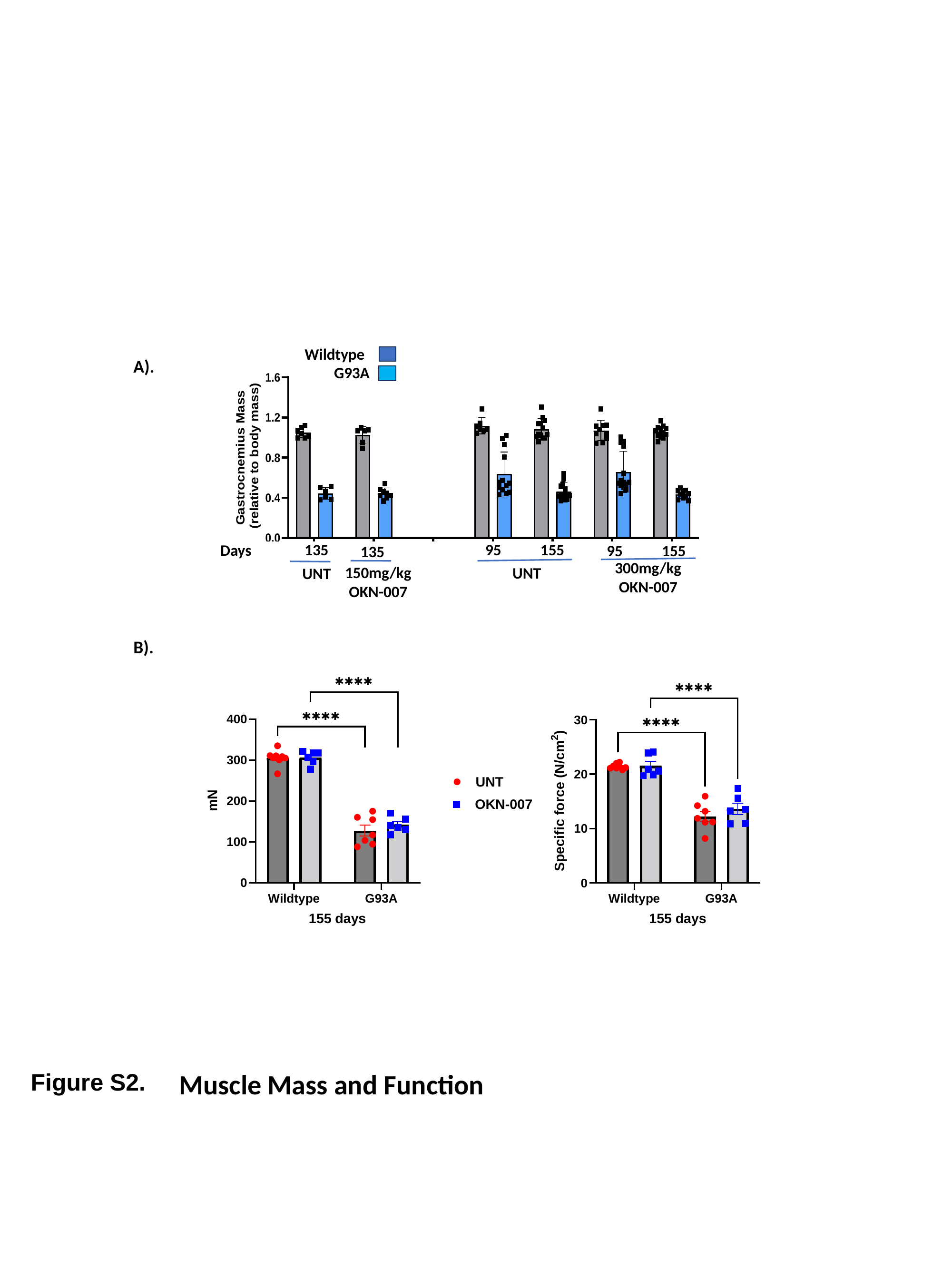

Wildtype
G93A
95 155
135
Days
95 155
135
300mg/kg
OKN-007
150mg/kg
OKN-007
UNT
UNT
A).
B).
Figure S2.
Muscle Mass and Function

## Slide 3
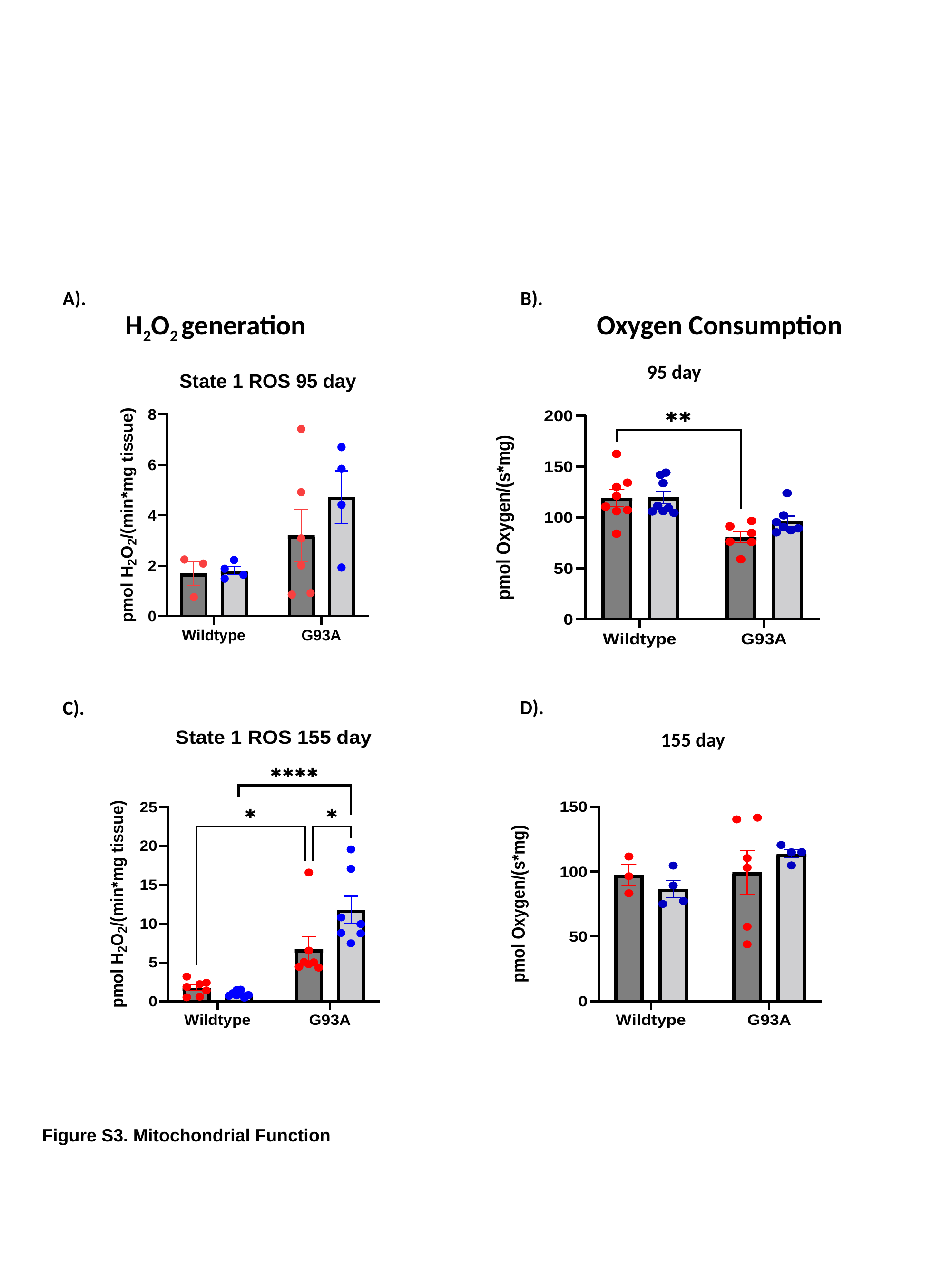

A).
B).
H2O2 generation
Oxygen Consumption
95 day
D).
C).
155 day
Figure S3. Mitochondrial Function

## Slide 4
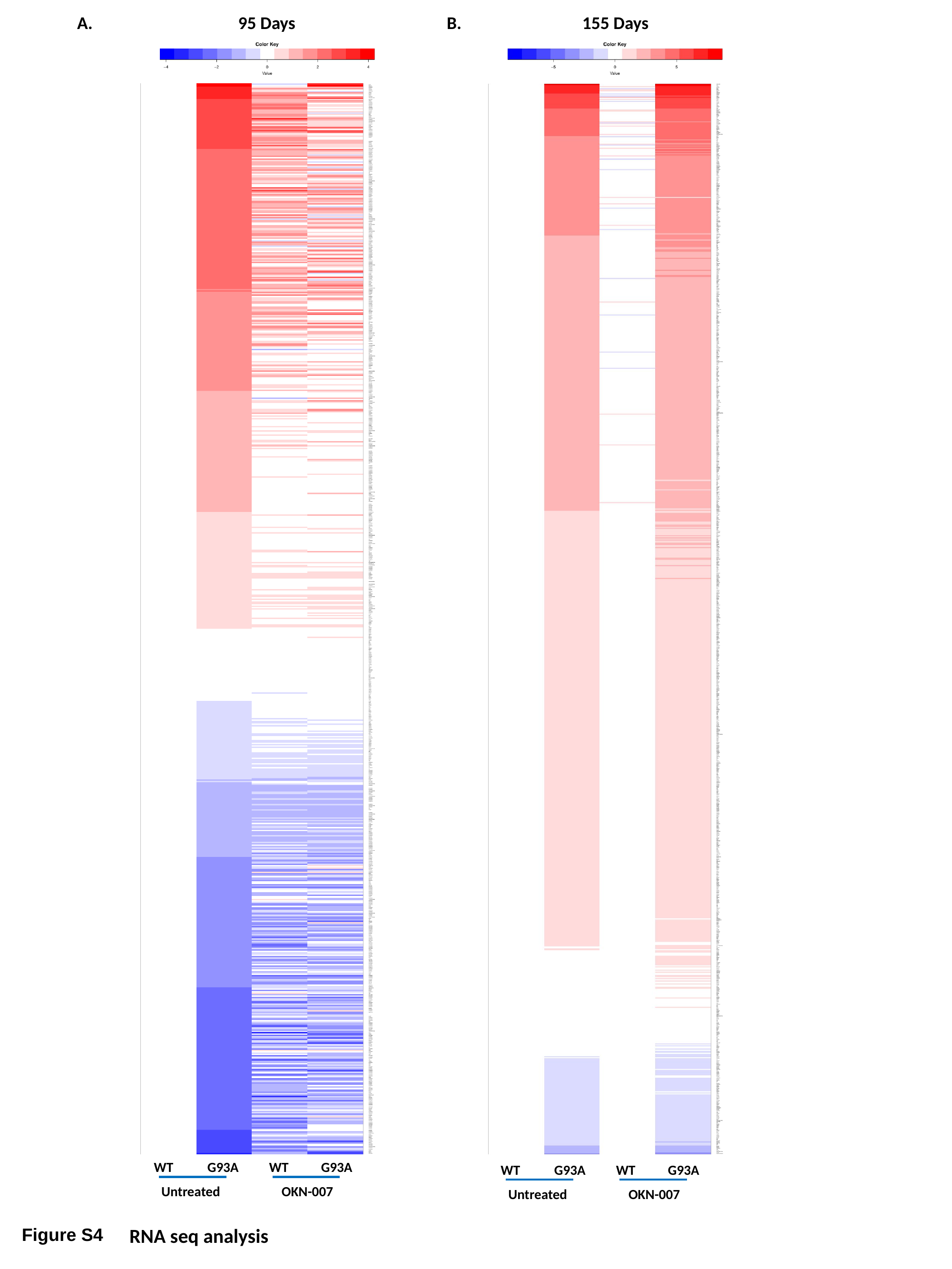

A.
95 Days
B.
155 Days
WT
G93A
WT
G93A
WT
G93A
WT
G93A
Untreated
OKN-007
Untreated
OKN-007
RNA seq analysis
Figure S4
